# Arabidopsis FIN219/JAR1 interacts with phytochrome A under far-red light and jasmonates in regulating hypocotyl elongation via a functional demand manner

**DOI:** 10.1371/journal.pgen.1010779

**Published:** 2023-05-22

**Authors:** Han-Wei Jiang, Kai-Chun Peng, Ting-Yu Hsu, Yen-Chang Chiou, Hsu-Liang Hsieh

**Affiliations:** 1 Institute of Plant Biology, College of Life Science, National Taiwan University, Taipei, Taiwan; 2 Department of Life Science, College of Life Science, National Taiwan University, Taipei, Taiwan; 3 Master Program in Global Agriculture Technology and Genomic Science, National Taiwan University, Taipei, Taiwan; Peking University, CHINA

## Abstract

Integration of light and phytohormones is essential for plant growth and development. FAR-RED INSENSITIVE 219 (FIN219)/JASMONATE RESISTANT 1 (JAR1) participates in phytochrome A (phyA)-mediated far-red (FR) light signaling in Arabidopsis and is a jasmonate (JA)-conjugating enzyme for the generation of an active JA-isoleucine. Accumulating evidence indicates that FR and JA signaling integrate with each other. However, the molecular mechanisms underlying their interaction remain largely unknown. Here, the *phyA* mutant was hypersensitive to JA. The double mutant *fin219-2phyA-211* showed a synergistic effect on seedling development under FR light. Further evidence revealed that FIN219 and phyA antagonized with each other in a mutually functional demand to modulate hypocotyl elongation and expression of light- and JA-responsive genes. Moreover, FIN219 interacted with phyA under prolonged FR light, and MeJA could enhance their interaction with CONSTITUTIVE PHOTOMORPHOGENIC 1 (COP1) in the dark and FR light. FIN219 and phyA interaction occurred mainly in the cytoplasm, and they regulated their mutual subcellular localization under FR light. Surprisingly, the *fin219-2* mutant abolished the formation of phyA nuclear bodies under FR light. Overall, these data identified a vital mechanism of phyA–FIN219–COP1 association in response to FR light, and MeJA may allow the photoactivated phyA to trigger photomorphogenic responses.

## Author summary

Interaction between plant growth and defense responses is critical for plant survival. However, the molecular mechanisms underlying their integration in response to environments remain elusive. Light provides plants with energy resources for photosynthesis and a signal for optimized growth and development. The phyA is the photoreceptor for perceiving far-red (FR) light in regulating different aspects of plant development. FIN219/JAR1 is a JA-conjugating enzyme that produces the physiologically active JA-isoleucine hormone, which triggers JA-regulated responses, including defense. Here, our findings reveal that phyA and FIN219 interact with each other, exogenous JA treatment enhances their interaction, and phyA-mediated FR light and FIN219-modulated JA signaling pathways are integrated in an antagonistic and mutually functional demand manner to regulate the photomorphogenic responses of seedling development, including inhibition of hypocotyl elongation, anthocyanin accumulation, chlorophyll content, and expression of light/JA-responsive genes. This regulatory relationship between both proteins may link plant growth and defense trade-offs upon exposure to various stresses.

## Introduction

The integration of light and various hormone signals dramatically affects plant growth and development [1–3]. The study of their interactions has been intensive. In particular, the interaction between light and jasmonates (JAs), a collection of jasmonic acid and its derivatives, has been attractive [3–7]. Light is the energy source for plant photosynthesis and also a signal for modulating plant development [8].

Plants contain different photoreceptors to sense various light wavelengths, including red (R)/far-red (FR) light perceived by phytochromes and blue light by cryptochromes [9]. These photoreceptor-mediated signaling pathways are involved in plant growth and development, from seed germination to flowering [10,11]. In contrast, JAs also play a vital role in regulating plant development, including the inhibition of hypocotyl elongation and primary roots, enhancement of lateral and adventitious roots, and modulation of reproductive development [12–14]. They also have substantial roles in plant defense and stress responses [15–17]. Thus, the interaction of light and JA is critical for plant development and survival, which is of many benefits to crop improvement.

Arabidopsis has five phytochromes (phyA to phyE) to perceive the R and FR light [18]. The phytochromes have overlapping and specific functions for controlling light-mediated physiological responses, leading to the adaptation of plant development to environmental cues [19]. PhyA is the only photoreceptor for FR light perception and can be activated by stable transfer from the cytoplasm to the nucleus under FR light [20–22]. This transfer allows interaction with negative regulators (e.g., phytochrome interacting factors (PIFs)) for their degradation and leads to the photomorphogenic development of seedlings [23–25].

Alternatively, phyA may interact directly with CONSTITUTIVE PHOTOMORPHOGENIC 1 (COP1), a master repressor of photomorphogenesis, under FR light [26]. This interaction allows positive regulators, such as HY5, to bind to the promoter of light-responsive genes for the photomorphogenic development of seedlings. Further evidence indicates that SUPPRESSOR OF PHYA-105 (SPA) proteins (SPA1 to SPA4) work with COP1 to negatively modulate HY5 levels under light conditions, including the dark, to regulate seedling development [27]. Studies have revealed that light-activated phyA interacts with SPAs to inhibit the interaction of COP1 and SPAs under FR light [28], which thus stabilizes the accumulation of transcription factors (e.g., HY5) for photomorphogenic development.

In addition to interacting with a significant number of light signaling components [29–31], phyA activated by FR light enters the nucleus from the cytoplasm via the assistance of Far-Red Elongated Hypocotyl 1 (FHY1) and FHY1-like 1 (FHL1), leading to the association with the nuclear bodies (NBs) in the nucleus [31,32]. Several signaling components, including HEMERA, affect the size and the number of phytochrome NBs, especially phyB NBs, in response to red light [26,33,34]. However, what factors affect the patterns of phyA NBs remains largely unknown. Currently, there are two types of phytochrome NBs. The first one is a transient phytochrome NB, which happens rapidly with small complexes upon exposure to light and then disappears 10–15 min later; the other type appears at 2–3 hours after red-light exposure and is more prominent and stable [35]. The emerging evidence indicates that the NBs may serve as the site for crosstalks between intrinsic and extrinsic signals and protein degradation. Besides, phyA interacts with components involved in various phytohormone signaling pathways [36,37]. However, the interaction between phyA and specific members in JA signaling remains unknown. Evidence has revealed the crosstalks between phyA-mediated FR light and JA signaling pathways [3,4,6,38]. Arabidopsis phyA is involved in regulating the JA-mediated degradation of JASMONATE-ZIM DOMAIN 1 (JAZ1) repressor proteins and the expression of *VEGETATIVE STORAGE PROTEIN* 1 (*VSP1*) as well as levels of 12-oxo-phytodienoic acid (OPDA) [4], an intermediate of JA biosynthesis. In addition, the Arabidopsis phytochrome chromophore mutants *hy1* and *hy2* showed elevated levels of JA and constant activation of COI1-dependent JA responses. Moreover, JA inhibits the expression of a group of light-inducible photosynthetic genes [39]. Thus, phytochrome chromophore-mediated light signaling and the JA signaling pathway may have a mutually antagonistic relationship. In addition, phytochrome inactivation by FR can strongly reduce a plant’s sensitivity to jasmonates [40]. Moreover, phyA in rice (*Oryza sativa*) requires jasmonate for photodestruction [41]. Further studies revealed that a JA-mediated increase in anthocyanin accumulation depended on phyA signaling under FR light [42]. Hence, phyA- and jasmonate-mediated signaling pathways are mutually regulated.

FAR-RED INSENSITIVE 219 (FIN219)/JASMONIC ACID RESISTANT 1 (JAR1) is a JA-conjugating enzyme responsible for the formation of a physiologically active JA-isoleucine (JA-Ile) [43]. FIN219 participates in phyA-mediated FR light signaling [44] and negatively regulates the level of COP1 via their direct interaction [45]. Moreover, FIN219 overexpression can trigger COP1 movement from the nucleus to the cytoplasm even in the dark, which leads to stable HY5 levels in the nucleus and photomorphogenic development of seedlings in darkness [45]. Therefore, it is crucial to tightly regulate FIN219 levels in Arabidopsis seedling development, which is consistent with our work showing that FIN219 levels in response to JA involve a negative feedback regulation, and FR light-regulated seedling development involves FIN219/JAR1-dependent and -independent pathways [6]. The mechanisms for the tight control of the FIN219 level in response to JA and FR light remain elusive.

To further elucidate the molecular mechanisms underlying phyA and FIN219 regulation under FR light and JA treatment, we used transgenic and molecular cell biology approaches to dissect their relationship. We found that phyA and FIN219 mutually regulate each other at the protein level by their direct interaction, which MeJA enhances under FR light and dark conditions. Intriguingly, phyA regulates the expression of JA-responsive genes in a FIN219-dependent manner, and conversely, FIN219 affects the expression of light-responsive genes in a phyA-dependent way. Thus, our data indicate that phyA and FIN219 act in a growth-defense tradeoff manner in Arabidopsis seedlings.

## Results

### The *phyA fin219* double mutant shows a synergistic phenotype under far-red light

**Fig 1:**
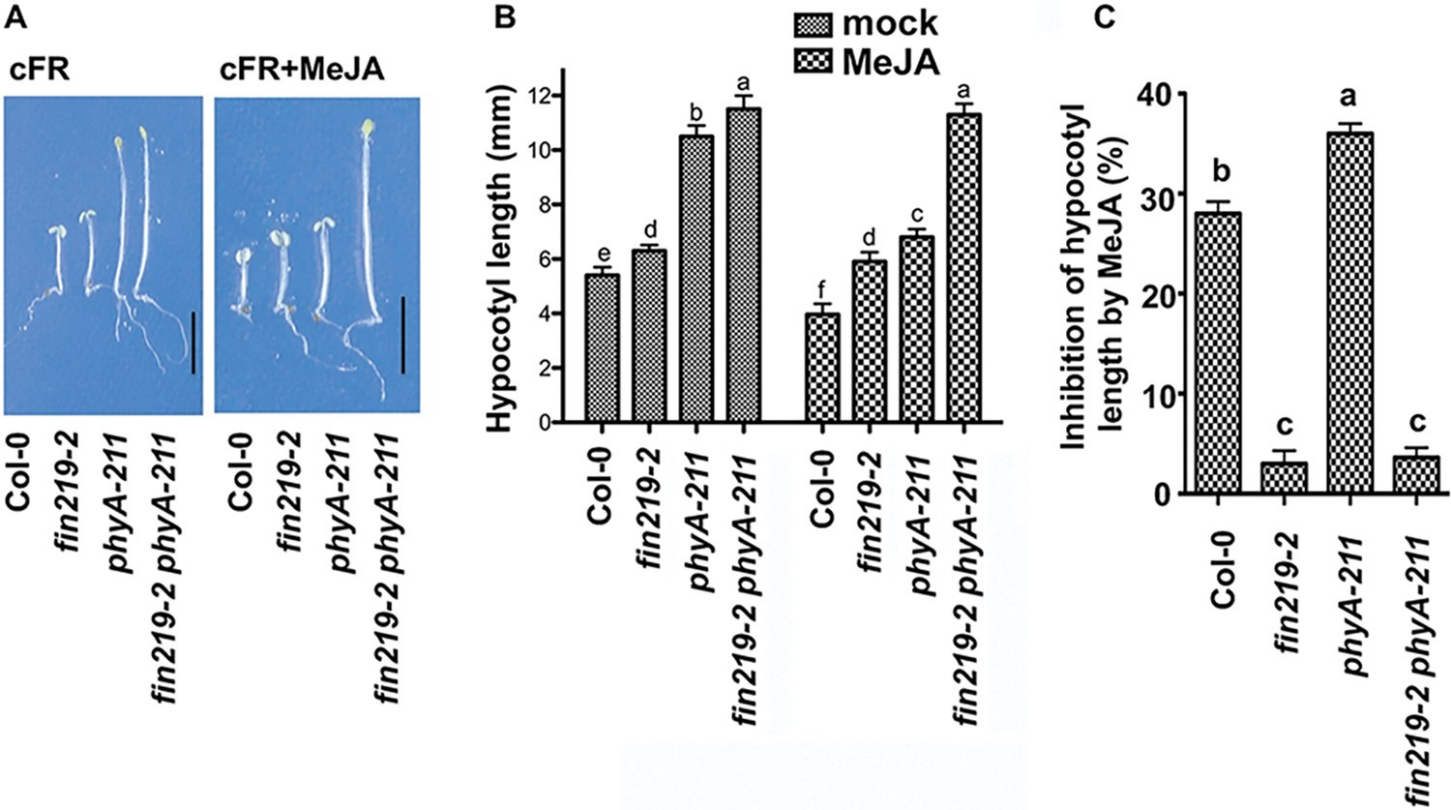
**The *fin219phyA* double mutants showed genetic interactions on exposure to light and MeJA to regulate hypocotyl elongation.** (**A**) Representative seedlings of the indicated genotypes grown under continuous far-red (cFR) light with or without 50 μM MeJA for three days. FR: 2 μmol m^-2^ s^-1^. Bars = 5 mm. (**B**) Quantitative measurement of hypocotyl lengths of seedlings shown in (**A**) (n = 30). (**C**) inhibition (%) of hypocotyl elongation of seedlings by MeJA shown in (**A**). Data are mean ± SE of three biological replicates. Different lowercase letters represent significant differences by ANOVA at P < 0.05.

Phytochrome A (phyA) is the only photoreceptor for the perception of FR light, and its mutant shows a long-hypocotyl phenotype under FR light [46,20,21,47,48]. The *fin219* mutant also has a long hypocotyl under FR light [44]. To further understand their regulatory relationship, we generated the double mutant *fin219-2phyA-211*, which showed an even longer hypocotyl than *fin219-2* or *phyA-211* under FR light (Fig 1A and 1B). Thus, FIN219 and phyA may act in a parallel pathway or work together to regulate hypocotyl elongation. In addition, exogenous MeJA treatment shortened hypocotyl for Col-0 but not for *fin219-2* and *fin219-2phyA-211* under FR light (Fig 1A and 1B). Moreover, *phyA-211* showed hypersensitive response to MeJA-mediated inhibition of hypocotyl elongation compared to the wild-type Col-0 and *fin219-2* (Fig 1C). However, the *fin219-2phyA-211* was less sensitive, similar to *fin219-2* under the same condition (Fig 1C). Taken together, FIN219 and phyA may have a genetic interaction to modulate hypocotyl elongation under FR light and MeJA treatment.

### FIN219-regulated light-responsive gene expression requires functional phyA, and phyA negatively regulated JA-responsive gene expression depends on FIN219

**Fig 2:**
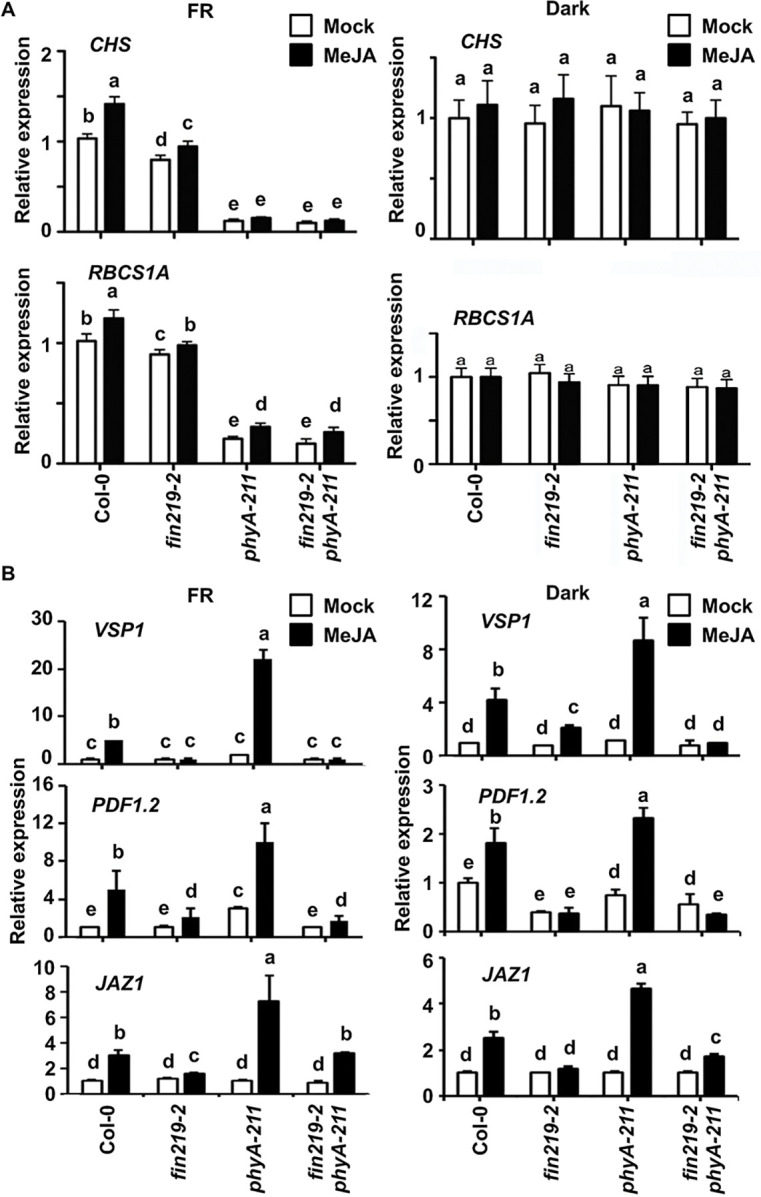
FIN219 and phyA regulate the expression of light- and JA-responsive genes, respectively, with a mutual requirement. (**A**) Quantitative real-time PCR (qRT-PCR) analysis of expression of light-responsive genes in seedlings grown under FR light or the dark. FR: 2 μmol m^-2^ s^-1^. (**B**) qRT-PCR analysis of expression of JA-responsive genes in seedlings grown under FR light or the dark. FR: 2 μmol m^-2^ s^-1^. Seedlings were grown on GM plates without or with 50 μM MeJA under FR light or the dark for 3 d. Actin 2 was an internal control. Data are mean±SE of three biological replicates. Different lowercase letters represent significant differences by ANOVA at P < 0.05.

To further elucidate the effect of *PHYA* and *FIN219* mutations on light and JA-responsive genes in integrating FR light and JA signaling pathways, we examined gene expression by quantitative real-time PCR (qRT-PCR). The single mutants *phyA* and *fin219-2* and double mutant *fin219-2phyA-211* were grown in FR or the dark with or without MeJA. Light-responsive genes, *CHS* and *RbcS*, were downregulated in *phyA*, *fin219-2*, and *fin219-2phyA-211* compared to Col-0 under FR light with or without MeJA (Fig 2A). The FIN219 effect on the expression of light-responsive genes appeared to require functional phyA. The regulated patterns were similar for *RbcS* in the dark and light (Fig 2A, bottom panel) but not for *CHS* (Fig 2A, top panel).

In addition, the expression of JA-responsive genes, such as *VSP1*, *PDF1*.*2*, and *JAZ1*, was substantially low in Col-0, *fin219-2*, *phyA-211*, and *fin219-2phyA-211* under FR light. However, their expression significantly increased in *phyA-211* compared to Col-0 and other mutants in the presence of MeJA under FR light and dark conditions, but the *fin219-2phyA-211* abolished the upregulated effect (Fig 2B). Hence, phyA may negatively modulate JA-responsive gene expression in a FIN219-dependent manner.

### FIN219 and phyA antagonize each other, and MeJA sensitizes phyA level

**Fig 3:**
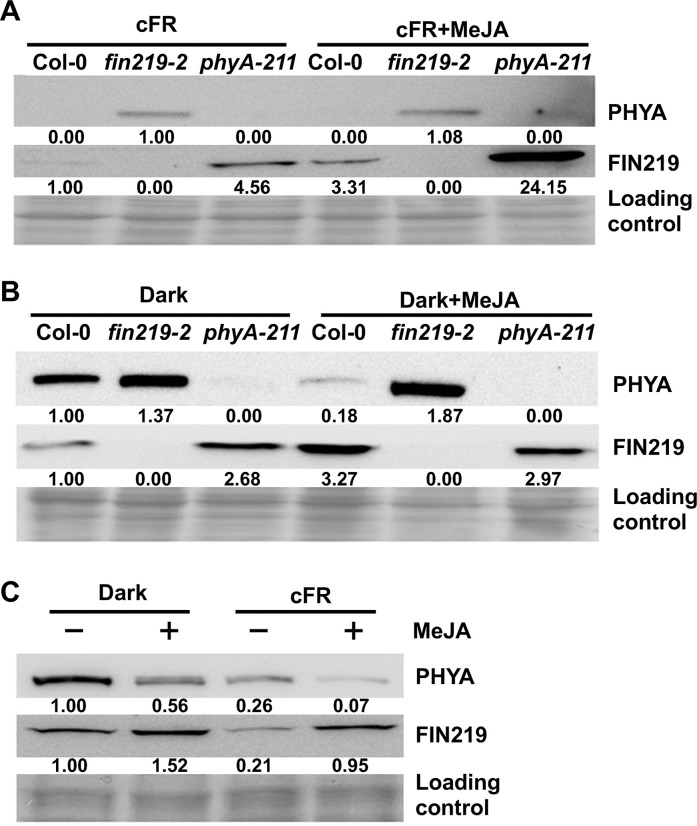
FIN219 and phyA antagonize each other under FR light and the dark. (**A**-**B**) Protein gel blot analysis of PHYA and FIN219 levels in Col-0, *fin219-2*, and *phyA-211* seedlings grown on plates without or with 50 μM MeJA under continuous FR (cFR) (**A**) and the dark (**B**) for 3 d. Each lane contains 70 μg total protein. Coomassie Brilliant Blue staining was a loading control. (**C**) Protein gel blot analysis of PHYA and FIN219 levels in Col-0 seedlings grown under the dark and FRc without (-) or with (+) 50 μM MeJA treatment for 3 d. Each lane contains 70 μg of total proteins. Coomassie Brilliant Blue staining was a loading control. The number below each blot represents the level of the indicated protein. The level of wild-type Col-0 was arbitrarily set to 1.

Genetic evidence and expression studies have revealed a particular regulatory relationship between phyA and FIN219. Moreover, phyA negatively regulates the expression of JA-responsive genes. We wondered whether MeJA would modulate the PHYA protein level under FR light conditions. Protein gel blot analyses indicated that PHYA protein level was upregulated in *fin219-2* compared to Col-0 under FR light and remained unchanged with MeJA compared to FR light alone (Fig 3A). In contrast, the FIN219 protein level was upregulated in *phyA-211* compared to Col-0 under FR light and significantly increased by MeJA in Col-0 and *phyA-211* (Fig 3A). These findings suggest that under FR light, FIN219 and phyA show an antagonistic relationship, but MeJA may suppress PHYA levels under FR. Likewise, *FIN219* and *phyA* mRNAs showed a similar pattern under FR light with or without MeJA (S1C and S1D Fig). In the dark, PHYA protein was abundant in Col-0 and substantially reduced by MeJA. However, MeJA significantly induced FIN219 protein compared to Col-0 in the dark alone (Fig 3B). Furthermore, FIN219 protein increased in *phyA-211* in the dark alone, but MeJA slightly reduced its level in *phyA-211* compared to Col-0 (Fig 3B). Thus, the positive effects of MeJA on FIN219 protein in the dark may require functional phyA, which differs from that in FR light. In the dark, levels of *FIN219* and *PHYA* mRNAs showed a similar regulatory relation upon exposure to MeJA (S1A and S1B Fig).

To further elucidate the JA effects on the PHYA and FIN219 protein levels in more detail, we used protein gel blot analyses with Col-0 seedlings grown under dark or FR light conditions with or without MeJA. MeJA downregulated the PHYA protein level in dark and FR light (Fig 3C). Conversely, the FIN219 protein level increased with MeJA treatment under both conditions (Fig 3C). MeJA could significantly increase *FIN219* and *PHYA* mRNA levels in the Col-0 in the dark and substantially decrease their mRNA levels under FR light (S1 Fig). Hence, FIN219 and phyA may involve different regulatory mechanisms at the transcript and protein levels in response to MeJA under dark and FR light conditions. MeJA likely activates FIN219 expression by downregulating the phyA protein level, thereby increasing the expression of JA-responsive genes.

### FIN219 and phyA, with a mutual requirement, participate in FR light- and MeJA-mediated inhibition of hypocotyl elongation

**Fig 4:**
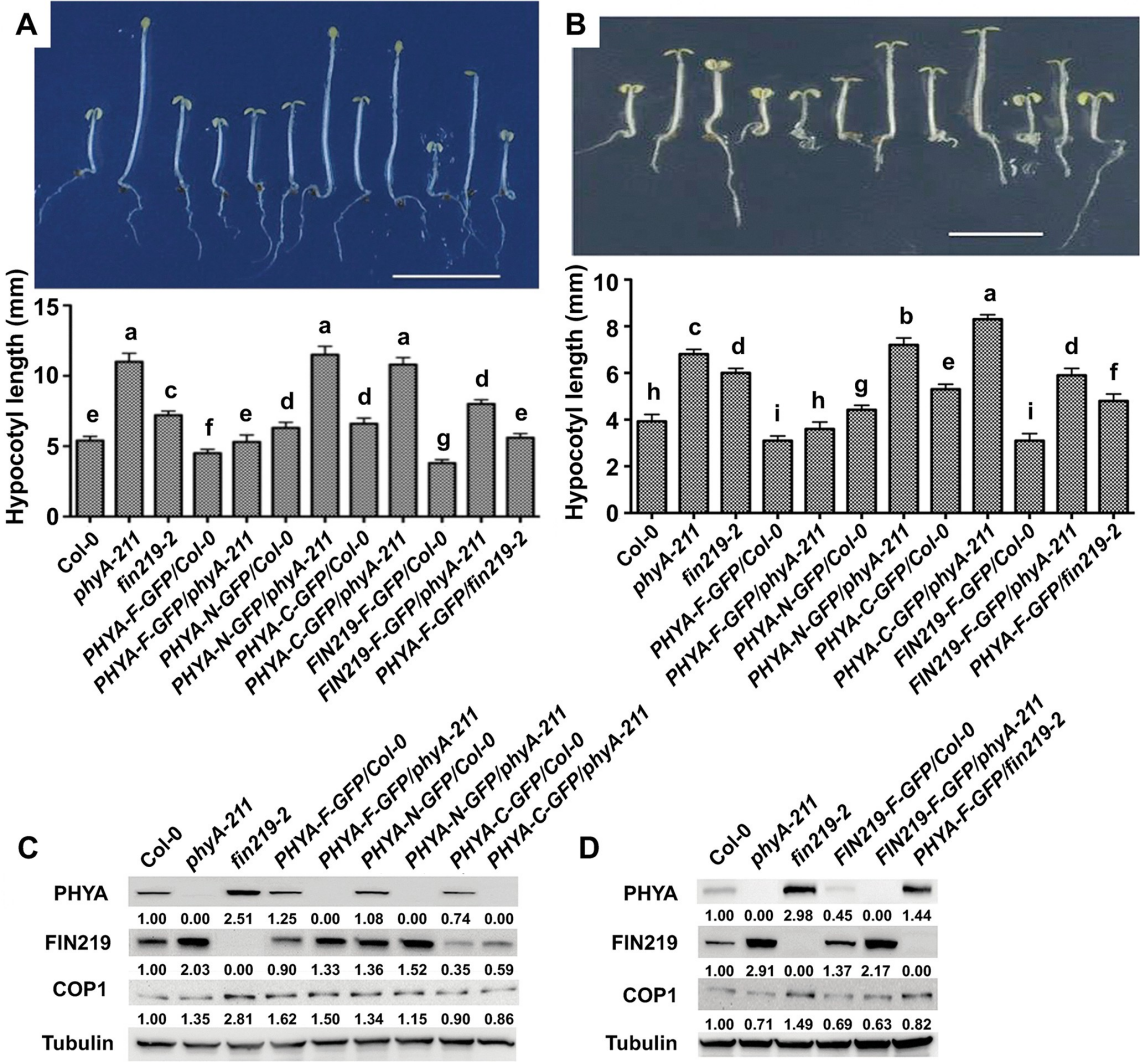
FIN219 and phyA show a mutually functional requirement to regulate hypocotyl elongation and an antagonistic relation for their protein levels under FR light and MeJA treatment. (**A**)The phyA required functional FIN219 to regulate FR light-mediated inhibition of hypocotyl elongation and vice versa. The seedlings of wild-type Col-0, various mutants, and transgenic lines shown in the figure were grown under FR light for three days and subjected to phenotypic examination and imaging (Above). Bar = 10 mm. The bottom panel quantifies hypocotyl lengths of the seedlings shown above. Data are mean ± SE for three experiments. Different lowercase letters represent significant differences by ANOVA at P < 0.05. (**B**)The phyA required functional FIN219 to regulate MeJA-mediated inhibition of hypocotyl elongation under FR light and vice versa. The seedlings of wild-type Col-0, various mutants, and transgenic lines shown in the figure were grown under FR light with 50 μM MeJA treatment for three days and subjected to phenotypic examination and imaging (Above). Bar = 5 mm. The bottom panel quantifies hypocotyl lengths of the seedlings shown above. Data are mean ± SE for three experiments. Different lowercase letters represent significant differences by ANOVA at P < 0.05. (**C**-**D**) FIN219 and phyA are antagonistic in response to FR light with MeJA treatment. Protein gel blot analysis of PHYA, FIN219, and COP1 levels in seedlings shown in (B). Each lane contains 70 μg total protein. Tubulin is a loading control. The number below each blot represents the level of the indicated protein. The level of wild-type Col-0 was arbitrarily set to 1.

In addition, we generated *FIN219* overexpression in Col-0 and *phyA-211* backgrounds (*FIN219-F-GFP/Col-0* and *FIN219-F-GFP/phyA-211*) and *PHYA* overexpression in Col-0 and *fin219-2* backgrounds (*PHYA-F-GFP/Col-0* and *PHYA-F-GFP/fin219-2*) transgenic lines for examining phenotypic responses and gene expression under FR light and FR light with MeJA treatment. We also confirmed all transgenic lines used in this study under FR light (S2 Fig). *PHYA-F-GFP/Col-0* showed a slight hypersensitivity with shorter hypocotyls than Col-0 under FR light or FR light with MeJA treatment (Fig 4A and 4B). However, *PHYA-F-GFP/phyA-211* showed a phenotype comparable to Col-0 (Fig 4A and 4B). Overexpression of both the N- or C-terminal regions of PHYA in Col-0 (*PHYA-N-GFP/Col-0* and *PHYA-C-GFP/Col-0*) exhibited a more extended hypocotyl phenotype than Col-0 but shorter than *phyA-211* in response to FR light or FR light with MeJA treatment (Fig 4A and 4B). These data suggest that the N- or C-terminus of PHYA may regulate the response to FR light and MeJA treatment under FR light.

However, *PHYA-N-GFP/phyA-211* and *PHYA-C-GFP/phyA-211* showed much longer hypocotyls than *PHYA-N-GFP/Col-0* and *PHYA-C-GFP/Col-0* under FR light or FR light with MeJA treatment (Fig 4A and 4B). This result implies that FR light- and MeJA-mediated inhibition of hypocotyl elongation requires functional phyA. In contrast, phyA function in JA signaling also required functional FIN219, as shown by *PHYA-F-GFP/fin219-2* with longer hypocotyls than *PHYA-F-GFP/Col-0* (Fig 4B).

Furthermore, *FIN219-F-GFP/Col-0* resulted in a shorter hypocotyl than Col-0 under FR light or FR light with MeJA treatment (Fig 4A and 4B). However, *FIN219-F-GFP/phyA-211* showed a more prolonged hypocotyl phenotype than *FIN219-F-GFP/Col-0* (Fig 4A and 4B), suggesting that the FIN219 function in FR light- and MeJA-mediated inhibition of hypocotyl elongation requires phyA. Thus, phyA and FIN219 may work together to modulate FR light and MeJA-mediated responses such as inhibition of hypocotyl elongation under FR light.

### FIN219 and phyA act antagonistically in the control of seedling development

To further understand the molecular mechanisms underlying the mutual requirement of phyA and FIN219 in regulating MeJA-mediated inhibition of hypocotyl elongation under FR light, we examined the expression of PHYA and FIN219 proteins and JA- and light-responsive genes in seedlings shown in Fig 4B. Protein gel blot analyses revealed significantly increased levels of endogenous PHYA protein in *fin219-2* and *PHYA-F-GFP/fin219-2* (Fig 4C and 4D). Accordingly, PHYA decreased in *FIN219-F-GFP/Col-0* (Fig 4D). These results suggest that FIN219 negatively regulates PHYA levels in seedling response to FR light and MeJA treatment. Endogenous PHYA protein levels in *PHYA-F-GFP/Col-0*, *PHYA-N-GFP/Col-0*, and *PHYA-C-GFP/Col-0* remained comparable to Col-0 (Fig 4C). In contrast, FIN219 protein level was more remarkable in *phyA-211*, *PHYA-N-GFP/Col-0*, *PHYA-N-GFP/phyA-211*, and *FIN219-F-GFP/phyA-211* than in Col-0 under the same condition, but decreased in *PHYA-F-GFP/Col-0*, *PHYA-C-GFP/Col-*,*0* and *PHYA-C-GFP/phyA-211* (Fig 4C and 4D). These data suggest phyA suppresses the FIN219 protein level via its C-terminal domain. In addition, the COP1 level increased in *fin219-2* under FR light with MeJA treatment. However, the COP1 level in other samples remained comparable to Col-0 (Fig 4C and 4D).

### phyA negatively regulates the expression of JA-responsive genes likely through FIN219, and FIN219 positively modulates light-responsive genes in a phyA-dependent manner under FR light in the presence of MeJA

**Fig 5:**
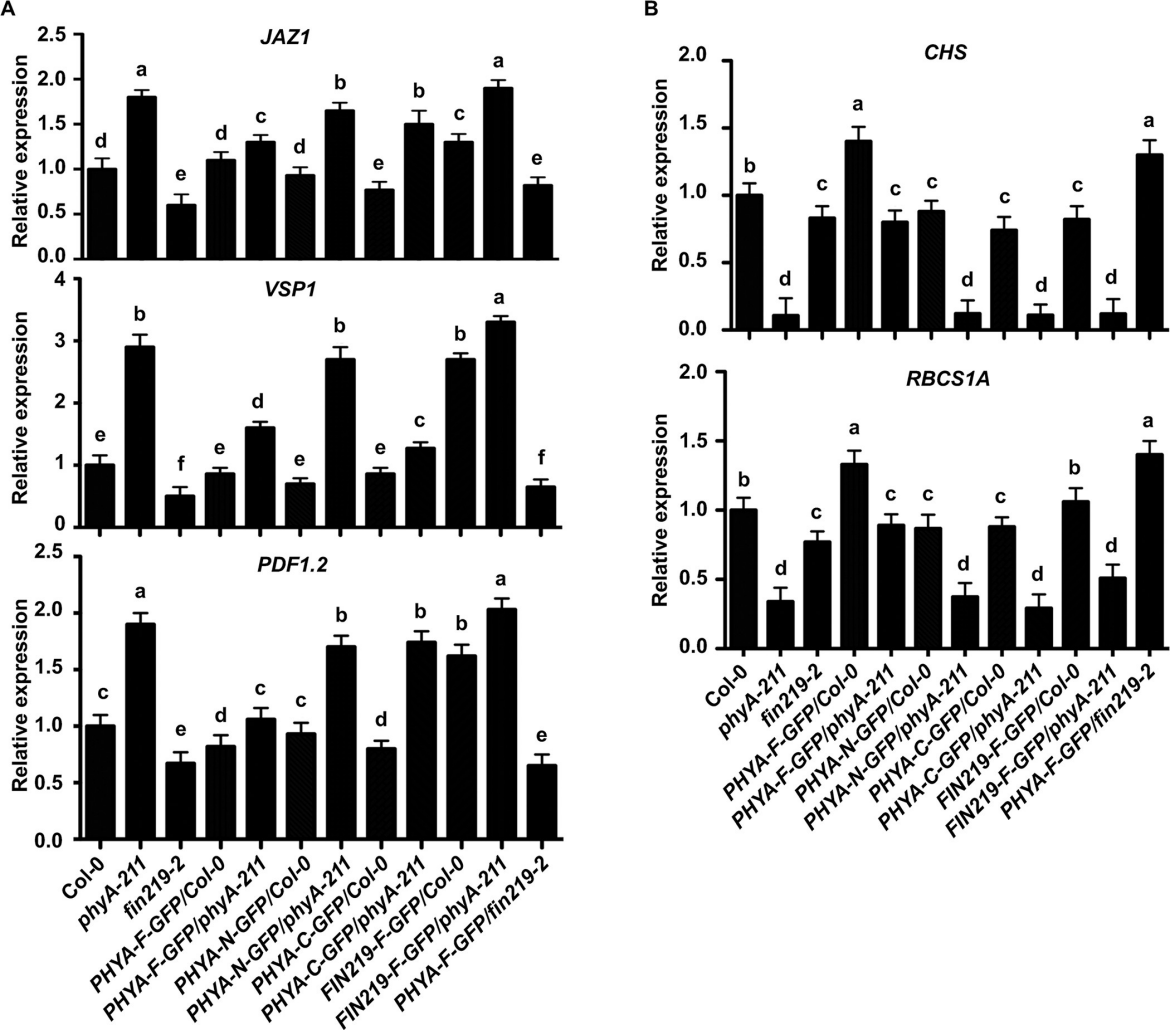
FIN219 and phyA regulate light- and JA-responsive genes, respectively, in a mutual-dependence manner in the presence of MeJA. (**A**) qRT-PCR analysis of the expression of JA-responsive genes in seedlings shown in Fig 4B. (**B**) qRT-PCR analysis of the expression of light-responsive genes in seedlings shown in Fig 4B. Actin 2 was an internal control. Data are mean ± SE for three experiments. Different lowercase letters represent significant differences by ANOVA at P < 0.05. FR light: 2 μmol m^-2^ s^-1^.

Further examination indicated that the expression of JA-responsive genes such as *JAZ1*, *VSP1*, and *PDF1*.*2* significantly increased in *phyA-211*, *PHYA-N-GFP/phyA-211*, and *PHYA-C-GFP/phyA-211* under FR light with MeJA treatment. Conversely, these JA-responsive genes vastly decreased in *PHYA-F-GFP/Col-0*, *PHYA-N-GFP/Col-0*, and *PHYA-C-GFP/Col-0* (Fig 5A). These data suggest that phyA negatively regulates JA-responsive gene expression under FR light. The expression of these JA-responsive genes reduced in *fin219-2* and substantially increased in *FIN219-F-GFP/Col-0* but decreased in *PHYA-F-GFP/fin219-2* compared to Col-0 (Fig 5A). Intriguingly, in *FIN219-F-GFP/phyA-211*, the expression of the examined JA-responsive genes was even higher than that in *FIN219-F-GFP/Col-0*. Hence, FIN219 positively regulated the expression of JA-responsive genes under FR light with MeJA treatment and phyA negatively, possibly through FIN219. In contrast, phyA positively modulated the expression of light-responsive genes, such as *CHS* and *RbcS1A*, under FR light with MeJA treatment, and FIN219 positively in a phyA-dependent manner under the same conditions (Fig 5B).

In addition to hypocotyl elongation, phyA regulates some MeJA-mediated physiological responses, including inhibiting chlorophyll content under white light, enhancing anthocyanin accumulation, and inhibiting seed germination under FR light in a FIN219-dependent manner (S3 Fig). Therefore, FIN219 and phyA participate in FR light and JA signaling pathways in a mutually practical demand manner, leading to control of seedling development.

### FIN219 interacts with phyA, and MeJA enhances their interaction

**Fig 6:**
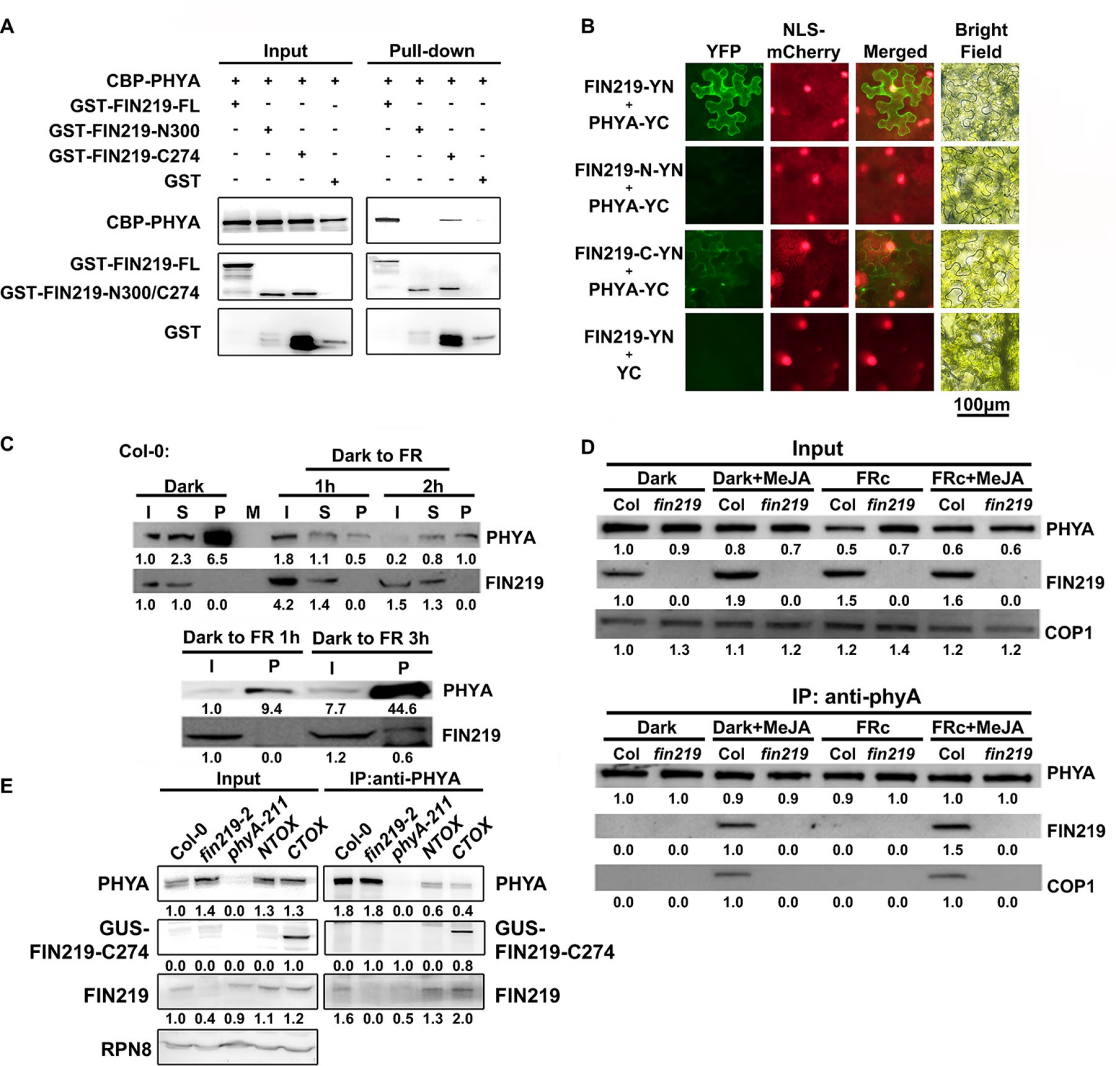
FIN219 physically interacts with phyA through its C-terminal region. (**A**) Pull-down assays indicated that the full-length and the C-terminal region of FIN219 interacted with PHYA. Recombinant proteins 1 μg purified from *E*. *coli* were subjected to pull-down assays. The GST-resin was used to pull down the GST-tagged proteins. (**B**) BiFC assays indicated that FIN219 interacted with PHYA in *N*. *benthamiana*. Both FIN219 and PHYA were expressed with the 35S promoter. After infiltration, the leaves were incubated for two days under white light and imaged after one-day FR light treatment. FR: 2 μmolm^-2^s^-1^. The signal was detected with a fluorescence microscope (Nikon H600L with DS-Ri2 camera). (**C**) Co-immunoprecipitation (Co-IP) assays of FIN219 and phyA interaction under FR light. Col-0 seedlings were grown in the dark for three days and then transferred to FR light at different times. Total proteins were immunoprecipitated by PHYA monoclonal antibodies and then probed with PHYA and FIN219 monoclonal antibodies. I: input proteins; S: supernatant after immunoprecipitation; P: pellets after immunoprecipitation; M: marker. (**D**) Co-IP assay of the FIN219 and phyA interaction under dark and cFR light conditions with 50 μM MeJA. The immunoprecipitation assay with PHYA antibody-bound beads uses an amount of 2 mg proteins extracted from 3-d-old Col-0 seedlings grown under the dark and FR light without or with 50 μM MeJA. The pellets were analyzed by immunoblotting with anti-PHYA, anti-FIN219, and anti-COP1 antibodies. cFR: 2 μmol m^-2^ s^-1^. (**E**) Co-IP assays revealed that FIN219 interacted with phyA through its C-terminal region. Total proteins were extracted from 3-d-old seedlings grown under FR light (2 μmolm^-2^s^-1^) and immunoprecipitated with PHYA antibodies. *NTOX*: *35S*: *GUS-FIN219-N300* and *CTOX*: *35S*: *GUS-FIN219-C274* transgenic lines in Col-0 background. The Western blots were probed with anti-PHYA, anti-GUS, and anti-FIN219 antibodies. The number below each blot represents the level of the indicated protein. The level of wild-type Col-0 was arbitrarily set to 1. Two biological repeats were performed with similar results; one representative result is shown here.

Because phyA and FIN219 proteins antagonize each other in response to FR light and MeJA treatment (Figs 3, 4C and 4D), we wondered whether the regulatory relationship between both proteins involved a direct interaction. We used yeast two-hybrid, pull-down, Bimolecular Fluorescence Complementation (BiFC), and co-immunoprecipitation (Co-IP) approaches to dissect their possible interaction. The yeast two-hybrid assay indicated that the full-length and C-terminal domain of PHYA, including the PAS and histidine kinase-related domain (HKRD), could interact with the full-length and N-terminal domain of FIN219 (S4A and S4B Fig). Further pull-down assays use recombinant proteins such as the full-length PHYA (CBP-PHYA), FIN219 (GST-FIN219-FL), and the N- and C-terminal regions of FIN219 (GST-FIN219-N300 and GST-FIN219-C274, respectively). The resulting outcomes showed that the full-length PHYA interacted with the full-length and the C-terminal region instead of the N-terminal region of FIN219 (Fig 6A). Further BiFC assays using tobacco leaves revealed that FIN219 interacted with PHYA via its C-terminal part but not the N terminus (Fig 6B). Moreover, FIN219 and PHYA interactions may occur in the cytoplasm under FR light (Fig 6B). Further Co-IP studies were proceeded by using wild-type Col-0 and *fin219-2* null mutant. With Col-0 seedlings grown in the dark and then transferred to FR light for 1, 2, or 3 h, Co-IP revealed that FIN219 did not interact with PHYA until 3 h exposure to FR light (Fig 6C). Exogenous MeJA could enhance FIN219 and PHYA interaction even in the dark and after short exposure to FR light (S5 Fig). Moreover, light-activated phyA interacted with SUPPRESSOR OF *phyA-105 1* (SPA1), disrupting the COP1–SPA1 complex and promoting photomorphogenesis [28]. Previous studies also showed that FIN219 interacted with COP1 under FR light [45]. By precipitating phyA from Col-0 seedlings grown in the dark and FR light with or without MeJA, phyA in Col-0, rather than *fin219-2*, could associate with FIN219 and COP1 only in the presence of MeJA under both the dark and FR light conditions (Fig 6D, bottom panel). Further Co-IP assays revealed that phyA in Col-0 did interact with FIN219 under FR light (Fig 6E, right panel). Moreover, phyA interacted with the C-terminal region of FIN219 (CTOX, overexpressing the C-terminal part of FIN219 in Col-0) rather than the N terminus of FIN219 (NTOX, overexpressing the N-terminal domain of FIN219 in Col-0) (Fig 6E). The N terminus of FIN219 and PHYA interaction in yeast two-hybrid assays may be relatively weak and transient. Taken together, phyA, FIN219, and COP1 can associate with each other under FR light in regulating seedling development.

### FIN219 and PHYA may regulate mutual subcellular localization

**Fig 7:**
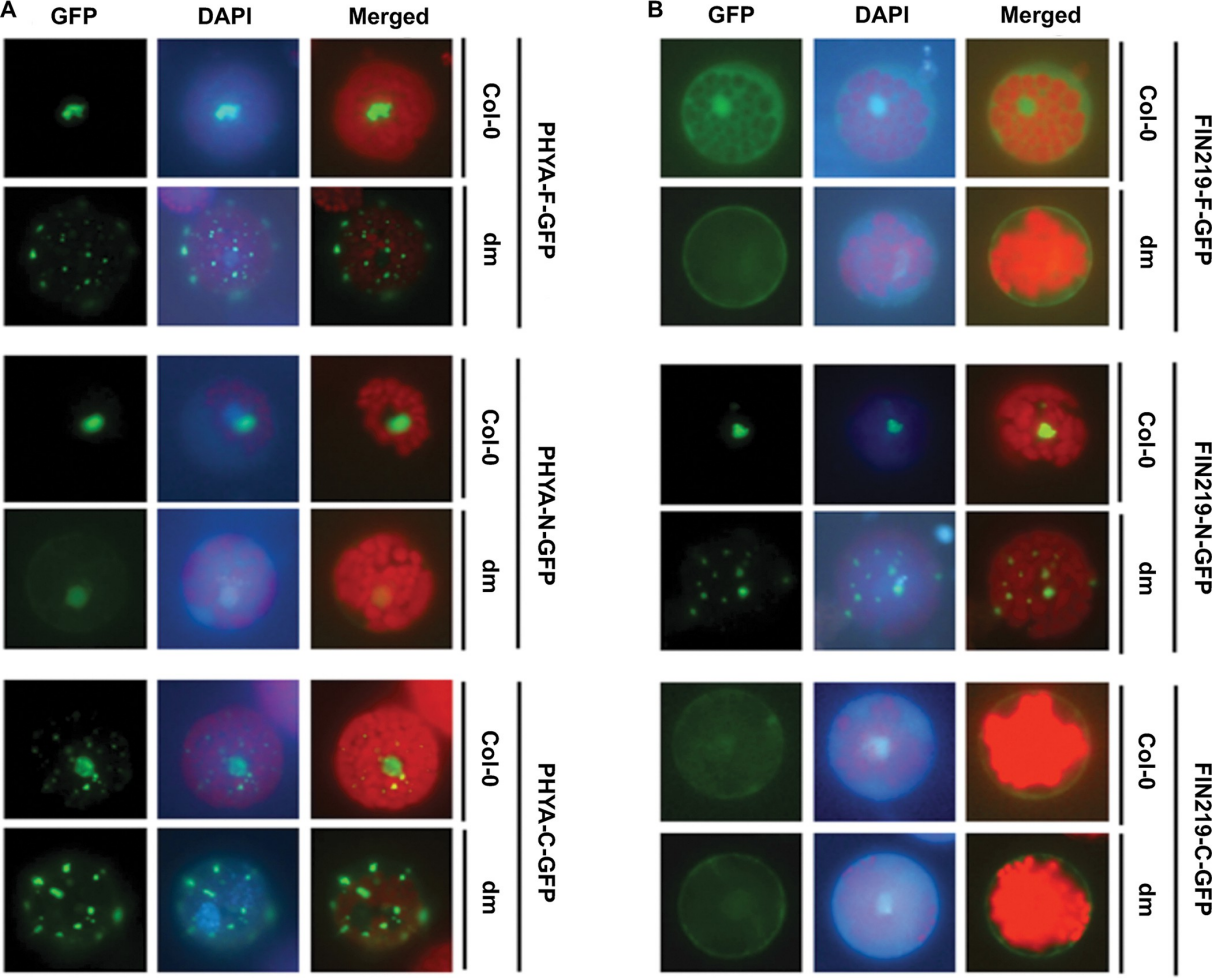
FIN219 and phyA regulate mutual subcellular localization under FR light. (**A**) FIN219 affected the subcellular localization of phyA under FR light. *PHYA-F-GFP*, *PHYA-N-GFP*, and *PHYA-C-GFP* constructs were introduced into the protoplasts isolated from Col-0 and *fin219-2phyA-211* double mutant (dm). (**B**) phyA affected the subcellular localization of FIN219 under FR light. *FIN219-F-GFP*, *FIN219-N-GFP*, and *FIN219-C-GFP* constructs were introduced into the protoplasts isolated from Col-0 and *fin219-2phyA-211* double mutant (dm). Images were taken after protoplasts were treated with 50 μM MeJA for 3 h under cFR (2 μmol m^-2^ s^-1^). Three biological repeats were performed with similar results; one representative result is shown here.

The phyA protein localizes in the cytoplasm in the dark and then translocates into the nucleus when FR light activates [22], and FIN219 is a cytoplasmic protein [44]. Previous studies indicated that phyA enters the nucleus via the assistance of the signal components FAR-RED ELONGATED HYPOCOTYL 1 (FHY1) and FHY1-Like (FHL) [32]. FIN219 genetically interacts with FHY1 under FR light [44]. We wondered whether FIN219 could affect the subcellular localization of phyA in response to FR light. The full-length PHYA or FIN219 fused with GFP (PHYA-F-GFP or FIN219-F-GFP), and the N-terminal and C-terminal domains of PHYA or FIN219 fused with GFP [PHYA-N- or -C-GFP or FIN219-N- or -C-GFP] were introduced into protoplasts of wild-type Col-0 or the double mutant *fin219-2phyA-211* under FR light. Indeed, PHYA-F-GFP in Col-0 was in the nucleus under FR light but dispersed in the cytoplasm of *fin219-2 phyA-211* (*dm*). PHYA-N-GFP was in the nuclear periphery in Col-0 and *dm* mutant under FR light. PHYA-C-GFP was in the cytoplasm and the nucleus in Col-0 and mainly in the cytoplasm in *dm* mutant (Fig 7A). FIN219-F-GFP was in the nucleus and the cytoplasm in Col-0. However, it was in the cytoplasm in *dm* mutant, FIN219-N-GFP was in the nucleus in Col-0 and the cytoplasm in *dm* mutant, and FIN219-C-GFP was in the cytoplasm in both Col-0 and *dm* mutant under FR light (Fig 7B). These data indicate that phyA and FIN219 may regulate a mutual subcellular localization response to FR light.

### FIN219 affects the formation of phyA nuclear bodies (NBs) under FR light

**Fig 8:**
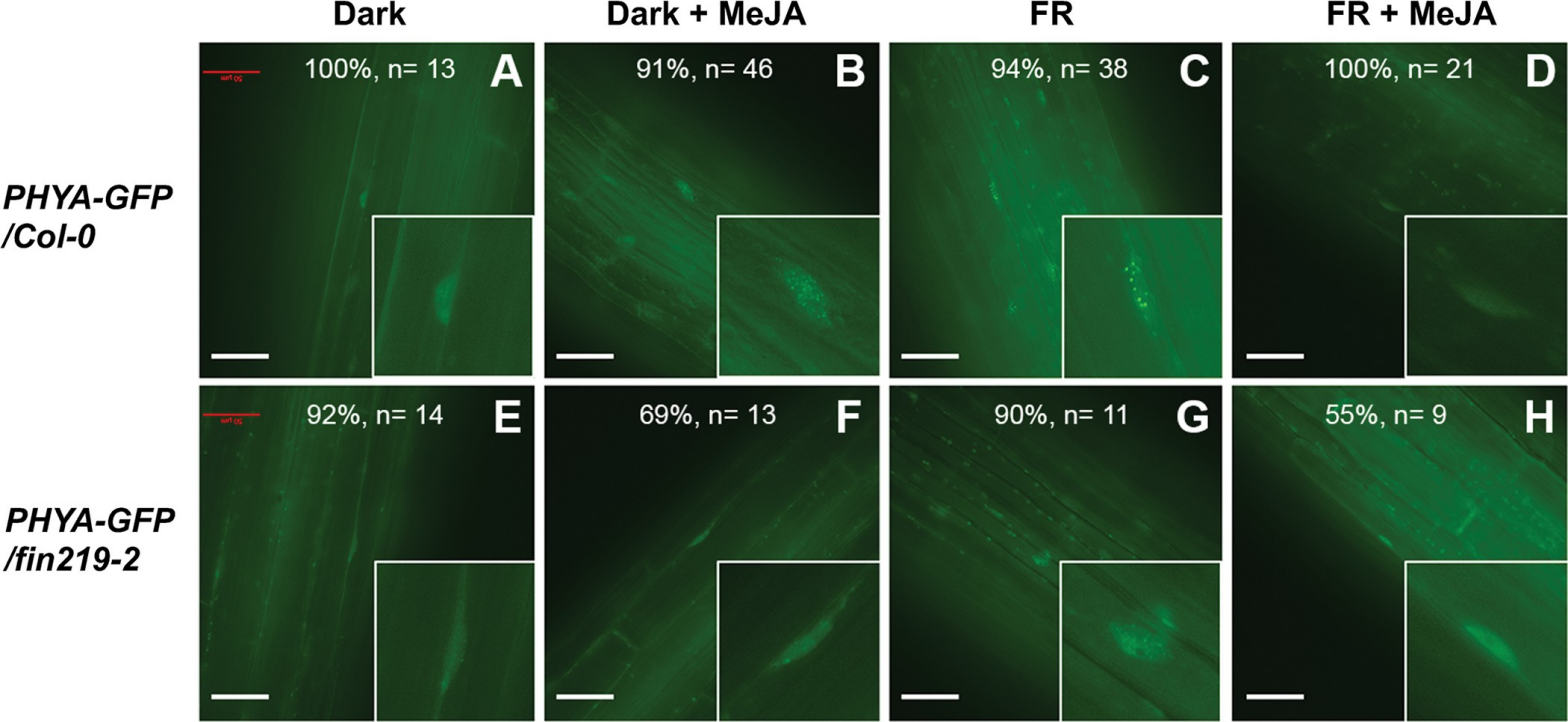
The formation of phyA NBs requires FIN219 under FR light. *PHYA-GFP/Col-0* (**A**-**D**) and *PHYA-GFP/fin219-2* (**E**-**H**) transgenic seedlings were grown on the MS medium under the dark (**A**, **B**, **E**, and **F**) or continuous FR light (**C**, **D**, **G**, and **H**) (2 μmol m^-2^s^-1^) in the absence (**A**, **C**, **E**, and **G**) or presence (**B**, **D**, **F**, and **H**) of MeJA (50 μM) for three days. The formation of phyA NBs in hypocotyl cells was examined with a Nikon H600L microscope. In each panel, the percentage indicates the 100x ratio of the cells with NBs patterns enlarged in the inset and total cells examined (= n). Scale bar: 50 μm.

We further used transgenic lines *PHYA-GFP/Col-0* and *PHYA-GFP/fin219-2* to examine phyA subcellular localization upon exposure to FR light and MeJA treatment. There was a PHYA-GFP signal in the nucleus of Col-0 but without the NB formation in the dark (Fig 8A), but MeJA addition resulted in phyA NBs under the darkness (Fig 8B). The PHYA-GFP signal with NBs appeared under FR light (Fig 8C). However, exogenous MeJA treatment under FR light abolished the phyA NBs (Fig 8D), which is an opposite effect compared to that in the dark (Fig 8B), suggesting that light regulates MeJA-mediated phyA NBs. In contrast, the darkness with MeJA treatment and FR light conditions significantly reduced the phyA NBs in the *fin219-2* mutant (Fig 8F and 8G). Taken together, these data suggest that FIN219 may regulate the formation of phyA-associated NBs in response to MeJA and FR light.

## Discussion

We report the phyA functions involved in JA signaling and its interaction with the JA-conjugating enzyme, FIN219/JAR1, to integrate FR light and JA signaling, leading to modulation of different physiological responses, including inhibition of hypocotyl elongation and anthocyanin accumulation. Interestingly, phyA and FIN219 suppress each other by their direct interaction at the protein level and also showed a functional requirement to regulate MeJA-mediated inhibition of hypocotyl elongation under FR light. Moreover, phyA and FIN219 affected their mutual subcellular localization in response to FR light and MeJA treatment, which likely led to the regulation of light- and JA-responsive genes. Surprisingly, the *fin219-2* mutation almost abolished phyA NBs under FR light. Our findings provide insights into understanding the crosstalk between FR light and JA signaling to regulate Arabidopsis seedling development.

The interaction between light and JA signaling regulates numerous physiological responses, including the inhibition of hypocotyl elongation, shade avoidance responses, secretion of extrafloral nectar, and plant defenses [49,5,38,3]. Previous studies found that Arabidopsis phytochrome chromophore mutants *hy1* and *hy2* showed no photoactive phytochromes and increased levels of JA and constitutive activation of COI1-mediated JA responses [39]. Moreover, exogenous MeJA suppressed the expression of *HY1* and several light-inducible genes [39]. Therefore, phytochrome chromophore-mediated light signaling and JA-triggered signaling appear to be mutually antagonistic. In addition, a *phyA*-null mutant, *phyA-211*, showed an elevated level of OPDA under dark and FR light conditions [4]. JA- and wound-induced JAZ1 degradation required functional phyA [4]. In addition, rice phyA requires JA for photodestruction [41]. JA results in phosphorylation of phyA at Ser598 [50], which likely reduces the interaction of phyA with its partners, such as NDPK2, and the phyA-mediated signaling pathway [51]. Therefore, JA can regulate phyA stability and activity. Here, we show that FIN219 and phyA work together to regulate hypocotyl elongation under FR light with or without MeJA treatment (Fig 1A and 1B). Moreover, we demonstrate that FIN219 and phyA interact with each other mainly in the cytoplasm under FR light with MeJA enhancement of their interaction through a practical demand manner to regulate hypocotyl elongation (Figs 6A–6D, 8 and S4). In addition, *PHYA-C-GFP/phyA-211* may result in more PHYA-C-GFP-FIN219 heterodimer formation than *PHYA-C-GFP/Col-0*, reducing FIN219 activity and more extended hypocotyl response to FR light and MeJA treatment. Therefore, phyA and FIN219 may play vital roles in integrating FR light and JA signaling to regulate seedling development.

Besides, exogenous MeJA significantly reduced the PHYA level in the dark and FR light, incredibly FR light. In contrast, MeJA significantly increased the FIN219 level (Fig 3C). MeJA treatment could lead to the production of JA in the cells, which can further result in increased levels of JA-Ile, likely due to the enhanced FIN219/JAR1 level, resulting in upregulated expression of JA-responsive genes and MeJA-mediated responses [52, Fig 3]. This result is consistent with the *FIN219*-overexpression transgenic line *FIN219-F-GFP/Col-0* that showed a significantly reduced PHYA level (Fig 4D), leading to a shorter-hypocotyl phenotype compared to Col-0 under FR light without or with MeJA treatment (Fig 4A and 4B). However, the level of FIN219 was greater in *FIN219-F-GFP/phyA-211* than *FIN219-F-GFP/Col-0*, but the former had a longer hypocotyl. All these results indicate that phyA and FIN219 suppressed each other with a mutually functional requirement to modulate hypocotyl elongation in response to FR light and MeJA. Moreover, the antagonistic relationship likely depends on FR light fluences due to the combined effects of signaling components such as FHY1 and COP1 in the phyA deficient mutant (S6 Fig). The molecular mechanisms underlying phyA and FIN219 mutual suppression may involve direct or indirect regulation such as posttranslational modification or SPA1 because FIN219 also negatively regulates *SPA1* gene expression [45].

Previous studies indicated that phyA was required for the degradation of JAZ1 on exposure to JA or wounding [4]. The *phyA* mutant showed decreased JA-mediated inhibition of root growth and less induction of *VSP1* by JA, which suggests that phyA acts as a positive regulator of JA-mediated responses. However, the OPDA content was more remarkable in *phyA-211* than Col-0 under FR light and dark conditions. In addition, the JAZ1 level was more significant in aerial parts and degraded in roots of *phyA-211* [4], suggesting that phyA might function as a negative regulator of JA biosynthesis in the aerial and have differential regulatory mechanisms of JA-mediated signaling in the aerial and underground parts of seedlings. This speculation is consistent with a report showing that shoots and roots have opposite metabolic responses to drought [53]. Here, we found that *phyA-211* showed changes in various MeJA-mediated physiological reactions (Figs 1 and S3), including MeJA-mediated inhibition of hypocotyl elongation under FR light (Fig 1) and chlorophyll content under white light (S3 Fig). However, the *fin219-2phyA-211* showed phenotypic responses similar to *fin219-2* in MeJA-mediated inhibition of hypocotyl elongation, chlorophyll content under white light, and seed germination (Figs 1C and S3). Thus, phyA might regulate some of the MeJA-mediated responses in a FIN219-dependent manner. This finding is consistent with the expression data indicating the negative modulation of JA-responsive genes, such as *VSP1*, *PDF1*.*2*, and *JAZ1*, by phyA dependent on functional FIN219 under both FR light and dark conditions (Fig 2B). However, FIN219 positively regulated the expression of light-responsive genes, including *CHS* and *RbcS1A*, in a phyA-dependent manner under FR light regardless of the presence of MeJA (Fig 2A). In addition, phyA negatively regulated *VSP1* expression under FR light and dark conditions with 50 μM MeJA treatment, which differed from a previous report [4] that showed phyA positively regulating *VSP1* expression under FR light with 20 μM MeJA and 2% sucrose. This discrepancy is likely due to the cross-effects of FR light intensity, sucrose, and MeJA concentrations in that high sucrose concentration will antagonize the light effect on the induction of light-responsive genes [54].

Upon activation, the translocation of phyA from the cytoplasm into the nucleus to modulate gene expression requires direct interaction with FAR-RED ELONGATED HYPOCOTYL1 (FHY1) [55] and its less abundant homolog, FHY1-LIKE (FHL) [31,32]. Genetic evidence indicated that FIN219 synergistically works with FHY1 to regulate hypocotyl elongation under FR light [44]. Our work showed FIN219 localized to the cytoplasm and nucleus and PHYA in the nucleus under FR light. However, FIN219 and phyA affected their mutual subcellular localization (Fig 7A and 7B). In particular, the N-terminal region of FIN219 was clearly in the nucleus, likely by physically interacting with PHYA (Figs 7B and S4B). However, the FIN219 C-terminus failed to enter the nucleus with functional phyA (Fig 7B). Its C-terminal domain alone may interact with other partners such as COP1 [45] and CRY1 [56], leading to restriction of the associated complex in the cytoplasm. In contrast, most of the C-terminal domain of PHYA was in the nucleus, which suggests that PHYA also requires its N-terminus, by interacting with FHY1, to enter the nucleus under FR light efficiently. Thus, FIN219 might interact with phyA and FHY1 or FHL to translocate into the nucleus in response to FR light and MeJA. In addition, the photoactivated phyA by FR light enters the nucleus with the formation of NBs through FHY1 and FHL leading to photomorphogenesis of seedling development [31,32]. Previous studies indicated that *phyA-302* mutants carrying a missense mutation (Glu to Lys) at amino acid 777 in the PAS2 domain of the C-terminal region of phyA resulted in a defect in HIR but not in VLFR [57]. The *phyA-302* showed longer hypocotyls and less angle between cotyledons than wild-type Col-0 under FR light. Moreover, *phyA-302* mutation abolished the NBs formation upon exposure to FR light [57], which suggests that the dimerization domain covering the amino-acid residue 777 of phyA is likely involved in the formation of NBs and highly correlated with reduced photomorphogenesis of the *phyA-302* mutant under FR light. The N-terminal domain of FIN219/JAR1 interacted with the C-terminal region of PHYA in yeast two-hybrid assays (S4A and S4B Fig). Further pull-down, BiFC, and Co-IP studies confirmed that the FIN219 C-terminal region interacted with PHYA (Fig 6A, 6B and 6E). The FIN219 N-terminal region may weakly interact with PHYA or associate with other signaling components such as FHY1 or FHL1 to enter the nucleus. Intriguingly, FIN219/JAR1 and phyA mutually regulated their subcellular localization under FR light (Fig 7), which is consistent with the result that phyA NBs require functional FIN219 under FR light (Fig 8G). In addition, the subcellular localization of phyA in the dark appeared to be in the nucleus without NBs formation (Fig 8A), which may be due to the association of FIN219 and phyA (Fig 6D), and FIN219 and FHY1 interaction, leading to the complex PHYA-FIN219-FHY1 localized in the nucleus under dark conditions although FHY1 preferentially interacts with Pfr phyA under red light [31]. Another regulator, HEMERA, identified as a suppressor of *PBG* (*PHYB*::*GFP* overexpression line) containing large NBs, was shown to regulate phyB NBs [34]. Its mutation increased small phyB NBs and led to a long-hypocotyl phenotype compared to wild-type Col-0 under red and FR light [34]. HEMERA affects phyA-mediated VLFR and HIR-FR responses as well. Strikingly, HEMERA negatively regulated the stability of phyA, PIF1, and PIF3 under red light [34]. HEMERA may likely control phyA NBs upon exposure to FR light. Besides, HEMERA/pTAC12 positively modulated chloroplast development by interacting with pTAC14 [58]. FIN219, as a JA-conjugating enzyme, also affects chlorophyll content and regulates chloroplast development under white light. Whether FIN219 and HEMERA work together to hold phyA NBs remains elusive.

Regulation of FIN219 and phyA in response to FR light and MeJA seems complex. They suppressed each other under dark and FR light conditions and FR light with MeJA treatment (Fig 3A and 3B). However, phyA positively modulated the FIN219 level in the dark with MeJA treatment (Fig 3B), which suggests that MeJA alters the regulatory patterns between FIN219 and phyA in cells. Moreover, MeJA treatment positively regulated *FIN219* and *PHYA* mRNA levels in the dark but not under FR light (S1 Fig). The enzyme protochlorophyllide reductase A (PORA) converts protochlorophyllide (Pchlide) to chlorophyllide, leading to chlorophyll biosynthesis. PhyA may participate in the inactivation of *PORA* expression under FR light. A rice JA-deficient mutant *hebiba* accumulated more Pchlide than wild-type in darkness [59], which suggests that JA represses Pchlide accumulation in darkness. JA-mediated suppression of Pchlide level in darkness is likely acting through phyA and FIN219 by enhancing the *PORA* gene, leading to chlorophyllide accumulation that will give rise to chlorophyll production upon exposure to light. In contrast, in FR light, MeJA-mediated reduction of phyA was likely due to changes in the ratio of different isoforms of phyA (phyA’ and phyA”) [Fig 3A and 3C; 59], which led to increased expression of *PORA* and chlorophyll greening rapidly on exposure to white light. In addition, phyA, MeJA, and FR light fluences regulated FIN219 abundance (Figs 3, S6). Thus, the regulation between phyA and FIN219 at the transcript and protein levels in response to MeJA under dark and FR light conditions might have different and complex mechanisms, which remain elusive.

PhyA interacts with many signal components to trigger signal transduction pathways [60,36,61]. We found that FIN219 interacted with phyA under prolonged exposure to FR light (Fig 6C), and exogenous MeJA could enhance FIN219, phyA, and COP1 interactions under FR light and in the dark (Fig 6D), suggesting that FIN219 associates with Pr or Pfr phyA in the presence of MeJA. A previous report indicated that the Pr form of phyA in the cytoplasm has a physiological function [62]. The physiological meaning underlying MeJA-mediated interaction between FIN219 and phyA remains elusive. However, our studies here revealed that FIN219 and phyA suppress each other through direct interaction via a mutual demand to regulate hypocotyl elongation. Moreover, FIN219 interacted with COP1 [45]. Under FR light and dark conditions, FIN219 interacted with phyA in the cytoplasm likely by binding with phosphorylated phyA because MeJA treatment can result in phyA phosphorylation at Ser598 [50]. Moreover, phosphorylation at Ser598 of phyA would reduce the binding affinity with its interacting partners [51]. FIN219 overexpression or exogenous MeJA could exclude COP1 from the nucleus to the cytoplasm [45] and enhance FIN219, phyA, and COP1 association in the cytoplasm (Fig 6B and 6D). The other pool of dephosphorylated phyA would associate with FHY1/FHY3 to trigger FR signal transduction in the nucleus, leading to the photomorphogenic development of Arabidopsis seedlings [54].

**Fig 9:**
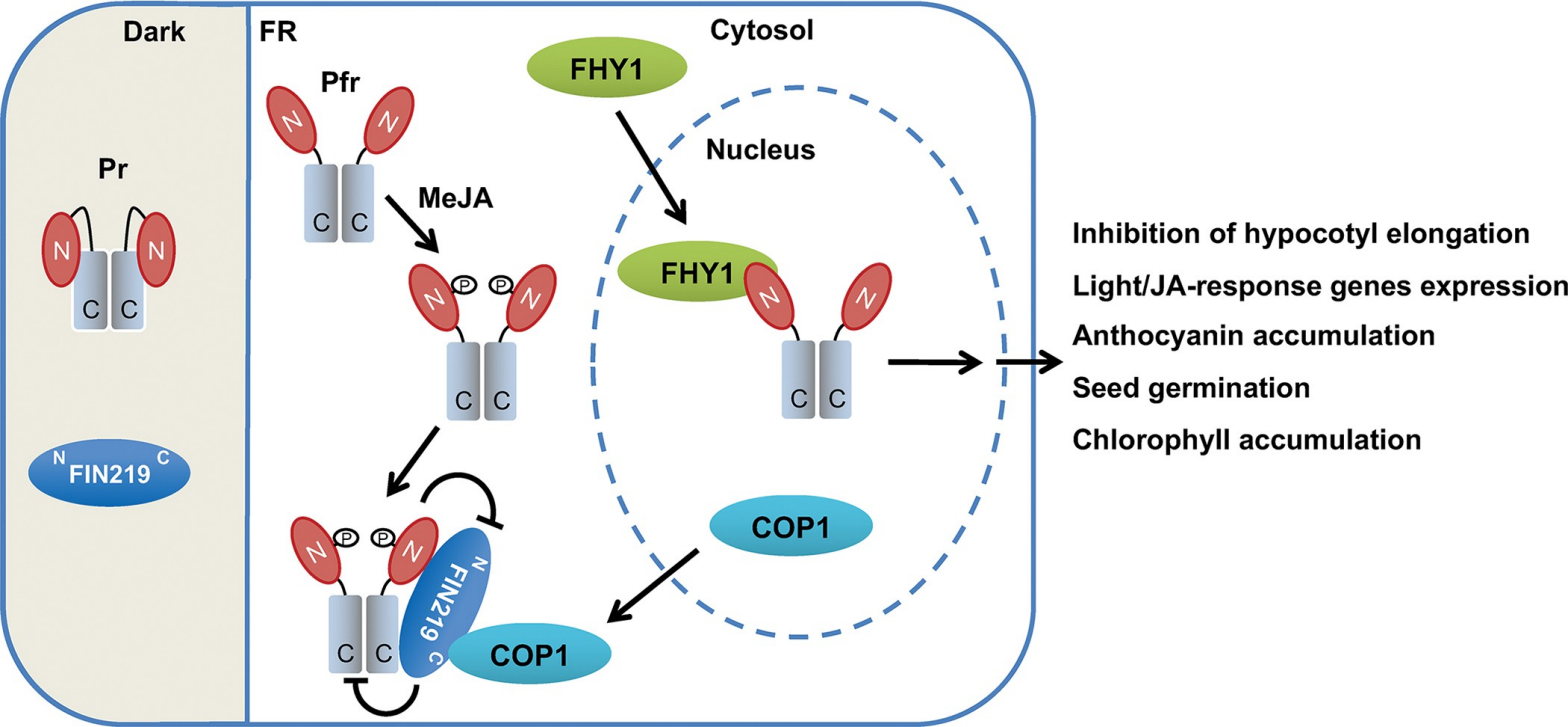
A model illustrates the functions of FIN219 and phyA in integrating FR light and JA signaling. FIN219 and the Pr form of phyA are in the cytoplasm in the darkness. FR light and MeJA can enhance the association of phyA and FIN219 as well as COP1 in the cytoplasm, likely leading to an interaction between the remaining phyA with light signaling components such as FHY1 for nuclear entrance and signal transduction. The result is photomorphogenic development, including inhibition of hypocotyl elongation, expression of light and JA-responsive genes, anthocyanin accumulation, and chlorophyll accumulation. Arrows indicate promoted action; inverted T represents a negative effect.

In conclusion, our work has revealed essential mechanisms of FIN219/JAR1 function in integrating phyA-mediated FR light and JA signaling. FIN219, as a JA-conjugating enzyme, can suppress the phyA level and activity by their direct interaction and regulate phyA subcellular localization, and vice versa in a functional demand manner. Thus, FR light and JA can enhance the association of FIN219/JAR1, phyA, and COP1 in the cytosol, which likely leads to an interaction between photoactivated phyA and light signaling components, including FHY1, for nuclear entrance and signal transduction. This signal transduction would promote the photomorphogenic development of seedlings and some JA-mediated responses such as anthocyanin accumulation and chlorophyll content in white light [Fig 9, 63].

## Materials and methods

### Plant materials and growth conditions

Throughout this study, the wild-type is the *Arabidopsis* Col-0 ecotype. We obtained the mutants *phyA-211* and *fin219-2* [45] in the Col-0 background from the Arabidopsis Biological Resource Center (ABRC). The *fin219-2phyA-211* double mutant was generated by crossing *fin219-2* with *phyA-211* and selecting homozygous plants from the F2 generation using T-DNA-specific primers described previously [45]. The full-length and different deletions of *PHYA* and *FIN219* coding sequences were inserted into pEarleyGate103 to generate *PHYA-F-GFP*, *PHA-N-GFP*, *PHYA-C-GFP*, and *FIN219-F-GFP* transgenic plants. The resulting plasmids were introduced into *Agrobacterium* GV3101 cells and then transformed into plants by the floral dip method [64]. Arabidopsis seeds were surface-sterilized for phenotype and molecular analysis. The plates were kept at 4°C in darkness for two days, transferred to white light at 22°C for 16 h, and then to specific light conditions for further experiments. We obtained the hypocotyl images using a Nikon digital camera (Nikon, Tokyo) and measured the hypocotyl lengths using ImageJ (http://rsb.info.nih.gov/ij/).

### Yeast two-hybrid assays

We conducted yeast two-hybrid experiments according to the Yeast Protocol Handbook (Clontech, USA). Bait and prey gene constructs were co-transformed into yeast (*Saccharomyces cerevisiae*) strain AH109. Protein-protein interactions in transformed yeast cells were assessed on synthetic defined (SD) agar medium without the amino acids His, Leu, Trp, and adenine. The selection medium contained X-α-gal as a marker, with galactose and raffinose as carbon sources. Yeast was streaked on X-α-gal plates, incubated at 30°C, and then examined for color development for another two days.

### Pull-down assays of recombinant proteins

We purified the FIN219 and PHYA recombinant proteins from *E*. *coli*. The *E*. *coli* cells containing various expression constructs, *CBP-PHYA*, *GST-FIN219-FL*, *GST-FIN219-N300*, and *GST-FIN219-C274*, were incubated in an LB medium for 16 hours. After 0.5 μM IPTG treatment, the proteins were purified by Camodulin Resin and Glutathione Sepharose 4B resin. For pull-down assay, 10 μl of the GST beads were mixed with 1 μg GST recombinant proteins overnight at 4°C in 1ml 1X PBS buffer and then added 1 μg CBP-PHYA protein for 2 hours at 4°C by rotating. After binding, the beads were washed with 1X PBS buffer eight times and centrifuged for 5 minutes at 4°C. The samples were boiled with 25 μl 6X SDS loading buffer for 15 minutes. For analysis, the samples were loaded on 10% SDS gel and detected with anti-GST and anti-phyA antibodies.

### Protein-protein interactions by co-immunoprecipitation (Co-IP) analysis

We ground the tissues from seedlings grown in the dark or cFR with or without 50 μM MeJA for three days with extraction buffer (50 mM Tris pH 7.5, 150 mM NaCl, 10 mM MgCl_2_, 0.1% NP-40, 1 mM phenylmethylsulfonyl fluoride, and 1x protease inhibitor). We performed Co-IP assays as described previously [45]. In brief, 2 mg proteins were mixed with beads and incubated at 4°C for 4 h, then washed three times with TBST washing buffer (1 X PBS with 0.05% Tween-20, pH7.5). We analyzed the pellets by SDS-PAGE and protein gel blot analyses.

### Protein gel blot assays

We extracted total proteins with grinding buffer (50 mM Tris pH 7.5, 150 mM NaCl, 10 mM MgCl_2_, 0.1% NP-40, 1 mM phenylmethylsulfonyl fluoride, and 1x protease inhibitor), mixed with a 6x SDS sample buffer, and heated in boiling water for 9 min before loading on 8% or 10% SDS-PAGE mini-gels. Gels were transferred to the PVDF membrane (Millipore, Billerica, MA) using an electrotransfer unit (Hoefer Pharmacia Biotech, USA). The blots were washed with PBST washing buffer (phosphate buffer and 0.1% Tween-20) three times for 5 min each, soaked in blocking solution (5% non-fat milk) for 5 min, and then probed with specific primary antibodies such as FIN219 and PHYA monoclonal antibodies [6; this study] and COP1 polyclonal antibodies [45] as well as PIF3 (AS163954, Agrisera)/4 (AS163955, Agrisera) polyclonal antibodies at 4°C overnight. The membranes were washed with PBST washing buffer twice and then incubated with an appropriate secondary antibody conjugated with horseradish peroxidase for 1 h. The signals were developed using the ECL system (T-pro LumiFast Plus ECL Kit) and detected using LAS-3000 (FUJIFILM).

### RNA preparation and quantitative real-time PCR

Total RNA was isolated using Rezol C&T (Protech Technologies). RNA (2 μg) was treated with DNase to prevent possible genomic DNA contamination and used as a template for cDNA synthesis with the ABI cDNA transcription kit (#4368814). Each PCR reaction contained 0.5 μg cDNA, 0.25 μM gene-specific forward/reverse primers, and 1XiQ SYBR Green supermix (Bio-Rad). We detected fluorescence signals with CFX96 Touch deep well real-time PCR detection system (Bio-Rad). Three-step PCR program was used: (1) 95°C, 3min. (2) 95°C, 10s; 60°C, 30s; 72°C, 30s. (40 cycles). (3) Program for melting curves. We analyzed all the quantification data with CFX Manager software (Bio-Rad). Gene-specific primers (S1 Table) were used for analyzing mRNA levels of *FIN219* (At2g46370), *VSP1* (At5g24780), *PDF1*.*2* (At5g44420), *JAZ1* (At1g19180), *CHS* (At5g13930), *RBCS-1A* (At3g18780), *PHYA* (At1g09570) and *ACTIN 2* (At3g18780) as an internal control to normalize the expression levels.

### Plant protoplast isolation and subcellular localization study

We analyzed transient expression in Arabidopsis protoplasts according to the Jen Sheen laboratory protocol [65]. Protoplast isolation and transfection were as described [45]. We used the GFP fusion constructs *p35S*: *PHYA-GFP* and *p35S*: *FIN219-GFP* for transfection. Nuclei were stained with 4′,6-diamidino-2-phenylindole (DAPI) staining solution (10 μg/ml DAPI in PBS). We observed the protoplasts with light microscopy (MZ16F; Leica) and used an RS Photometrics CoolSNAP camera (DFC490; Leica) to obtain images with the corresponding IM50 software.

### Bimolecular fluorescence complementation (BiFC) analysis

The *Nicotiana benthamiana* was incubated in the cabinet for four weeks under long-day conditions. We cloned full-lengths of FIN219 or FIN219-N or FIN219-C regions and PHYA coding sequences into pEarleyGate201-YFP^N^ and pEarleyGate202-YFP^C^, respectively [66] and transformed the constructs into *A*. *tumefaciens* C58C1 and transiently expressed in *N*. *benthamiana* by Agrobacterium infiltration. After the infiltration, the plants were incubated at 22°C under white light for two days and transferred to FR light for one day. We detected the signal with a fluorescence microscope (Nikon H600L with DS-Ri2 camera).

### Images of nuclear speckles formation of phyA

Seedlings of *PHYA-GFP/Col-0* and *PHYA-GFP/fin219-2* were grown on Murashige and Skoog (MS) medium containing ethanol or MeJA (50 μM) at 22°C under continuous dark or FR light **(**2 μmol m^-2^ s^-1^) conditions for three days. We observed nuclear speckles of hypocotyl cells with Nikon H600L microscopes.

### Chlorophyll extraction and quantification

Harvested seedlings were extracted with 100 μl of 80% acetone for 16 h at 4°C in the dark with vigorous mixing. We added an amount of 50 μl extract to 450 μl absolute alcohol and used a 200 μl sample for spectrophotometric analysis. Then we measured the absorbance at 660, 647, and 720 nm using Tecan Infinite 200 PRO (Tecan) with 96-well plates. Total chlorophyll content was determined according to the extinction coefficient calculated as described [67].

### Anthocyanin extraction and determination

We weighed harvested seedling samples, ground them with liquid nitrogen, and extracted the total plant pigments. The anthocyanin content was quantified using absorbance (A) at 530 nm and 657 nm with the Tecan Infinite 200 PRO (Tecan) and normalized to fresh weight. We determined the total anthocyanin content as described equation [23].

### Generation of PHYA Monoclonal antibody

PCR amplified a cDNA fragment corresponding to the coding region of PHYA with primers containing a built-in XhoI site and cloned it into the pCAL-n vector in-frame with an upstream CBP-tag. The CBP-PHYA- pCAL-n construct was introduced into the BL21 strain. The recombinant CBP-PHYA fusion protein was purified by 10% SDS-PAGE and electroeluted by Electro-Eluter (Model 422, Bio-Rad). We sent the eluted CBP-PHYA recombinant proteins to LTK BioLaboratories to produce monoclonal antibodies. The specificity of PHYA monoclonal antibodies was determined by using recombinant proteins of the full-length PHYA (CBP-PHYA-F), the N-terminal, and the C-terminal domains of PHYA (CBP-PHYA-N and CBP-PHYA-C), wild-type Col-0 and *phyA* mutants (S6 Fig).

### Statistical analysis

We conducted statistical analyses with IBM SPSS Statistics version 22. Growth and bioassay data were analyzed using one-way ANOVA with posthoc Tukey’s test to determine the statistically significant means (P<0.05). Different alphabets represent a statistically significant difference.

## Supporting information

S1 Fig MeJA can suppress FIN219 and PHYA transcript levels under FR light rather than dark conditions. (**A**-**B**) qRT-PCR analysis of *FIN219* (**A**) and *PHYA* (**B**) transcript levels in the indicated genotypes under dark. (**C**-**D**) qRT-PCR analyses of *FIN219* (**C**) and *PHYA* (**D**) transcript levels in the indicated genotypes under FR light (2 μmol m^-2^ s^-1^). Seedlings were grown under dark (**A**-**B**) or FR light (**C**-**D**) without or with 50 μM MeJA for 3 days. Actin 2 was an internal control. Data are mean ± SE of three experiments.
(TIF)

S2 Fig qRT-PCR analysis revealed that PHYA transcripts were highly expressed in different PHYA transgenic lines. The seedlings of wild-type Col-0 and various *PHYA* overexpression transgenic lines in Col-0 and *phyA-211* mutant backgrounds as shown in the figure were grown under FR light (2 μmol m^-2^ s^-1^) for 3 days, then used for total RNA extraction and subjected for qRT-PCR analysis. Actin 2 was an internal control. Data are mean ± SE of three experiments.
(TIF)

S3 Fig phyA was involved in regulating some MeJA-mediated physiological responses in a FIN219-dependent manner. (**A**) Chlorophyll content in the FR-blocked greening of indicated seedlings grown on GM plates under FR light (2 μmol m^-2^ s^-1^) without or with 50 μM MeJA for 3 days followed by white light for 1 day. (**B**) Anthocyanin content in seedlings grown on GM plates under FR light (2 μmol m^-2^ s^-1^) without or with 50 μM MeJA for 3 days. (**C**) Effect of MeJA on chlorophyll content of seedlings grown on GM plates without (Control) or with 5 μM MeJA treatment under white light (70 μmol m^-2^ s^-1^) for 7 days. (**D**) Effect of MeJA on the germination rate (%) of seeds sown on GM plates without (Control) or with 50 μM MeJA treatment under cFR light (2 μmol m^-2^ s^-1^) for 3 days. Data are mean ± SE of three biological replicates. Different lowercase letters represent significant differences by ANOVA at P < 0.05.
(TIF)

S4 Fig Yeast two-hybrid studies indicated that FIN219 interacted with phyA. (**A**)Schematic diagrams of five different deletions of PHYA apoprotein as prey showed PHYA interaction with FIN219 as bait through its C-terminal domain. (**B**) Schematic diagrams of three different deletions of FIN219 as prey showed FIN219 interaction with PHYA as bait through its N-terminal domain. Yeast cells were transformed with PHYA-AD and FIN219-BD, grown on selective medium, and exhibited β-galactosidase activity. PAS: Per/Arndt/Sim domain; HKRD: histidine kinase-related domain; AD: active domain; NC: N-terminal coiled-coil domain; CC: C-terminal coiled-coil domain; BD: binding domain.
(TIF)

S5 Fig Co-immunoprecipitation assays revealed MeJA enhancement of the FIN219-phyA interaction under the dark condition and FR light transition. Col-0 seedlings were grown in the dark with 50 μM MeJA for 3 d (**A**) and then transferred to FR light (2 μmol m^-2^ s^-1^) for different times (**B-C**). Total proteins 2 mg extracted from the seedlings were immunoprecipitated with phyA monoclonal antibodies, then probed with PHYA and FIN219 monoclonal antibodies. I: input proteins; S: supernatants after immunoprecipitation; P: pellets after immunoprecipitation.
(TIF)

S6 Fig FIN219 levels negatively regulated by phyA depend on FR light fluences. Protein gel blot analysis indicated that FIN219 levels in *phyA* mutant were differentially regulated by FR light fluences. The seedlings of Col-0, *phyA-211* and *fin219-2* were grown under different fluences of FR light for 3 days. The extracted proteins were subjected for Protein gel blot analysis. The primary antibodies were PHYA and FIN219 monoclonal antibodies. Tubulin was a loading control. The number below each blot represents the level of the indicated protein. The level of wild-type Col-0 was arbitrarily set to 1.
(TIF)

S7 Fig Gel blot analysis of PHYA detection specificity for monoclonal antibodies raised against PHYA protein. (**A**) Photograph of purified recombinant CBP-PHYA proteins. Purified recombinant proteins of the full-length and N-terminal and C-terminal domains of PHYA fused with a calmodulin binding peptide (CBP-PHYA-F, CBP-PHYA-N, and CBP-PHYA-C, respectively) were separated by 10% SDS-PAGE and stained with Coomassie blue dye (left panel). Protein gel blot analysis of recombinant CBP-PHYA proteins with different phyA monoclonal antibodies. PHYA monoclonal antibody #2 specifically recognized the full-length and N-terminus of PHYA (middle panel, with a longer running time of the gel) or PHYA monoclonal antibody #5 for the C-terminus of PHYA (right panel, with a longer running time of the gel). Dilution factor: 10,000X. (**B**) Protein gel blot analysis of PHYA protein detection specificity in seedlings of the indicated genotypes grown in the dark for 3 d. All *phyA* mutants were in a Columbia background. *phyA*: *phyA-1*; phyA-T: SALK_014575 mutants. Total protein (60 μg) was loaded in each lane and blots were probed with monoclonal antibody #2 against PHYA at dilution ratio 5000X.
(TIF)

S1 Table Primer pairs are used for quantitative real-time PCR. (TIF)

S2 Table Primer pairs are used for probe synthesis. (TIF)
