# Supplementary figures and images for: Arabidopsis FIN219/JAR1 interacts with phytochrome A under far-red light and jasmonates in regulating hypocotyl elongation via a functional demand manner
Source: PLoS Genet. 2023 May 22;19(5):e1010779. doi: 10.1371/journal.pgen.1010779 (PMC10237651; doi:10.1371/journal.pgen.1010779)

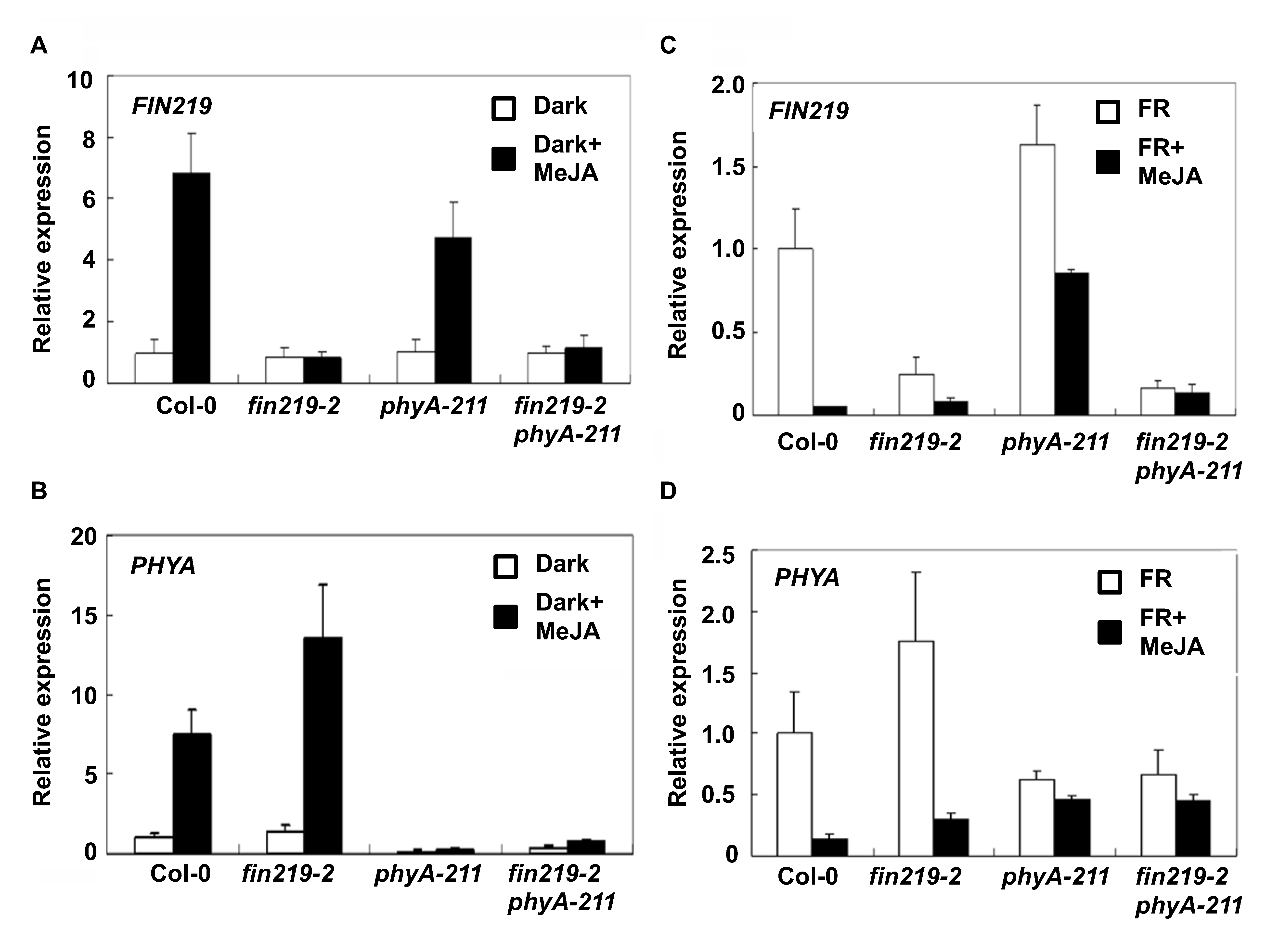

Supplement: S1 Fig — (A-B) qRT-PCR analysis of FIN219 (A) and PHYA (B) transcript levels in the indicated genotypes under dark. (C-D) qRT-PCR analyses of FIN219 (C) and PHYA (D) transcript levels in the indicated genotypes under FR light (2 μmol m-2 s-1). Seedlings were grown under dark (A-B) or FR light (C-D) without or with 50 μM MeJA for 3 days. Actin 2 was an internal control. Data are mean ± SE of three experiments. (TIF) [file pgen.1010779.s001.tif]

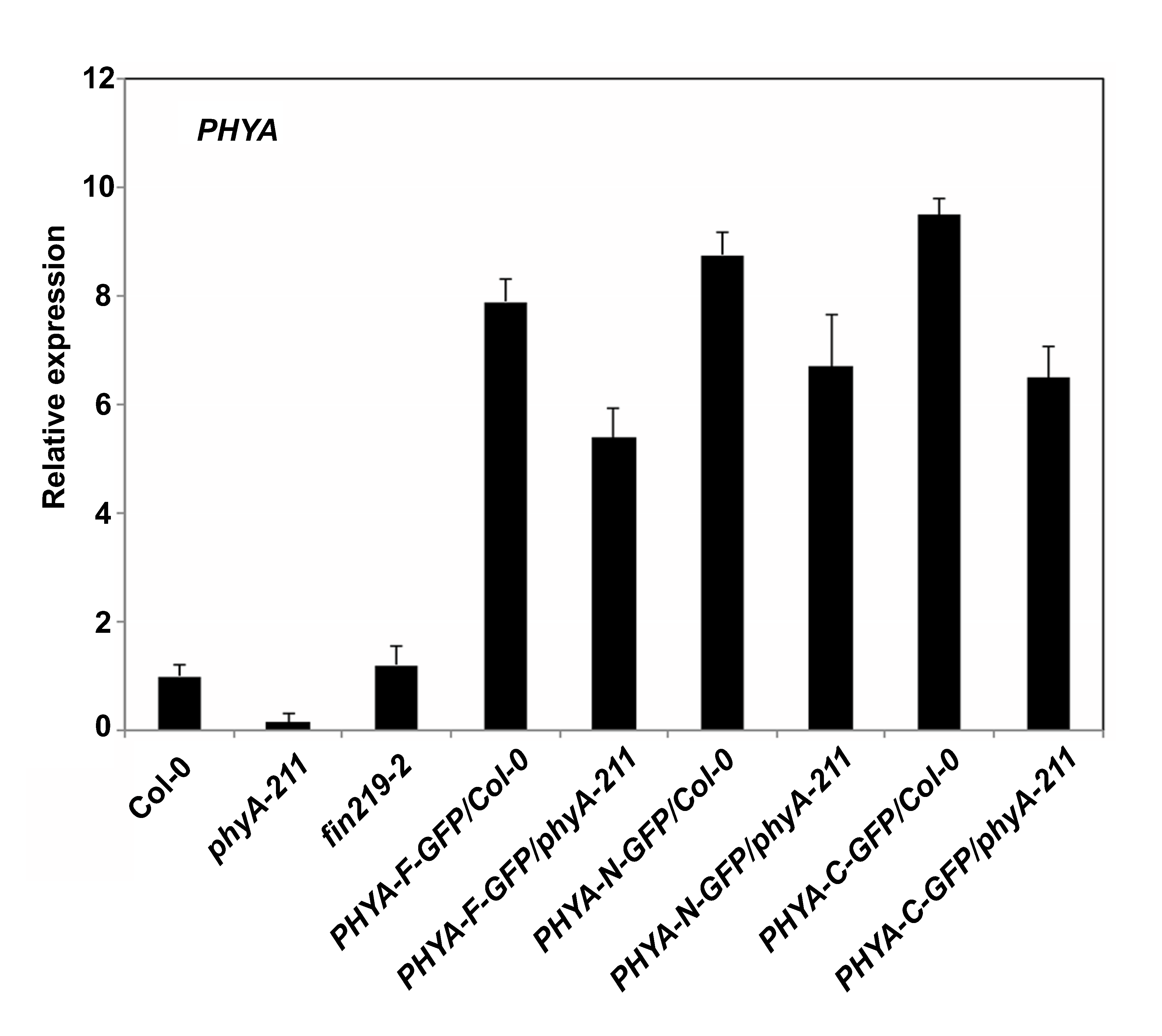

Supplement: S2 Fig — The seedlings of wild-type Col-0 and various PHYA overexpression transgenic lines in Col-0 and phyA-211 mutant backgrounds as shown in the figure were grown under FR light (2 μmol m-2 s-1) for 3 days, then used for total RNA extraction and subjected for qRT-PCR analysis. Actin 2 was an internal control. Data are mean ± SE of three experiments. (TIF) [file pgen.1010779.s002.tif]

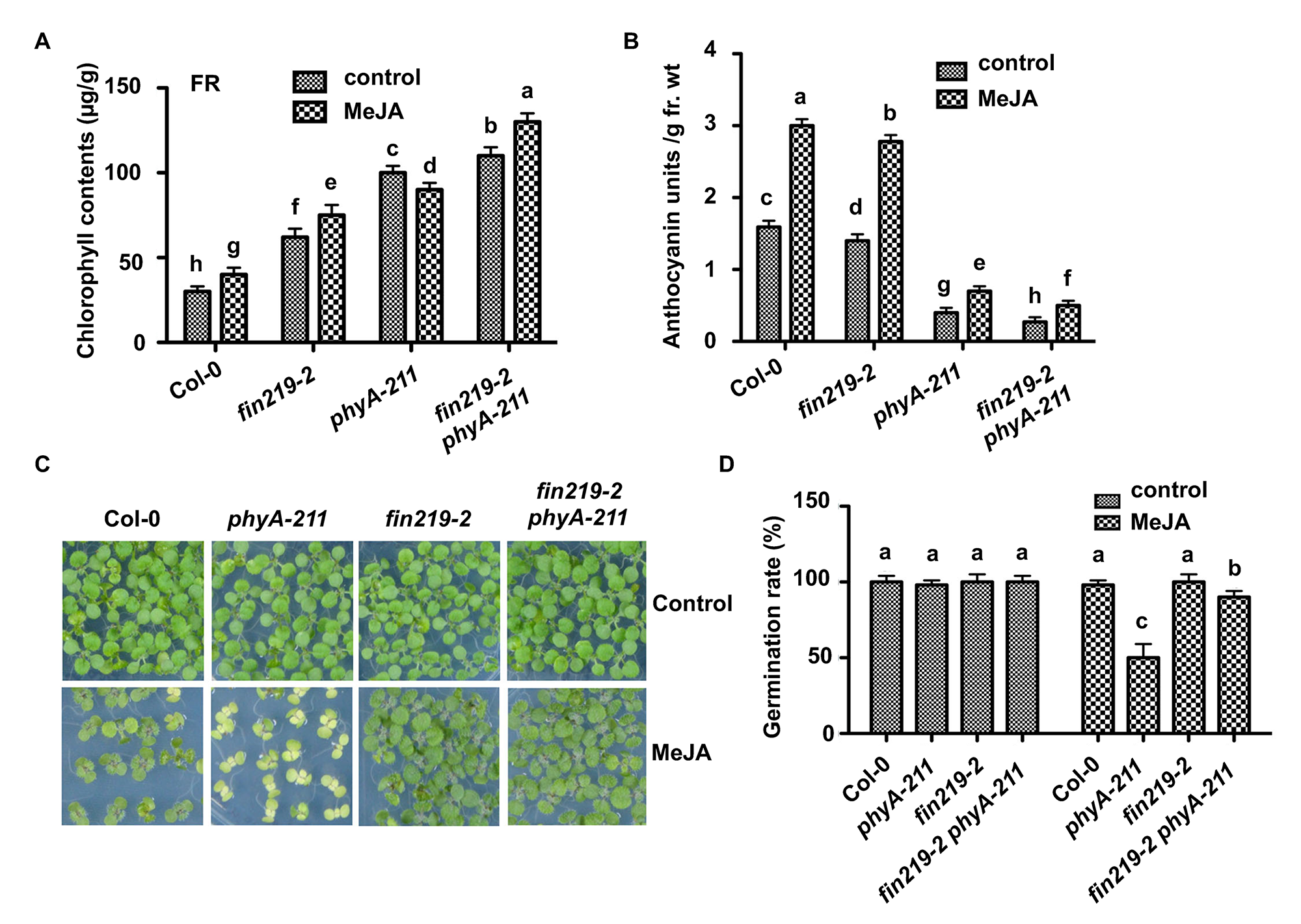

Supplement: S3 Fig — (A) Chlorophyll content in the FR-blocked greening of indicated seedlings grown on GM plates under FR light (2 μmol m-2 s-1) without or with 50 μM MeJA for 3 days followed by white light for 1 day. (B) Anthocyanin content in seedlings grown on GM plates under FR light (2 μmol m-2 s-1) without or with 50 μM MeJA for 3 days. (C) Effect of MeJA on chlorophyll content of seedlings grown on GM plates without (Control) or with 5 μM MeJA treatment under white light (70 μmol m-2 s-1) for 7 days. (D) Effect of MeJA on the germination rate (%) of seeds sown on GM plates without (Control) or with 50 μM MeJA treatment under cFR light (2 μmol m-2 s-1) for 3 days. Data are mean ± SE of three biological replicates. Different lowercase letters represent significant differences by ANOVA at P < 0.05. (TIF) [file pgen.1010779.s003.tif]

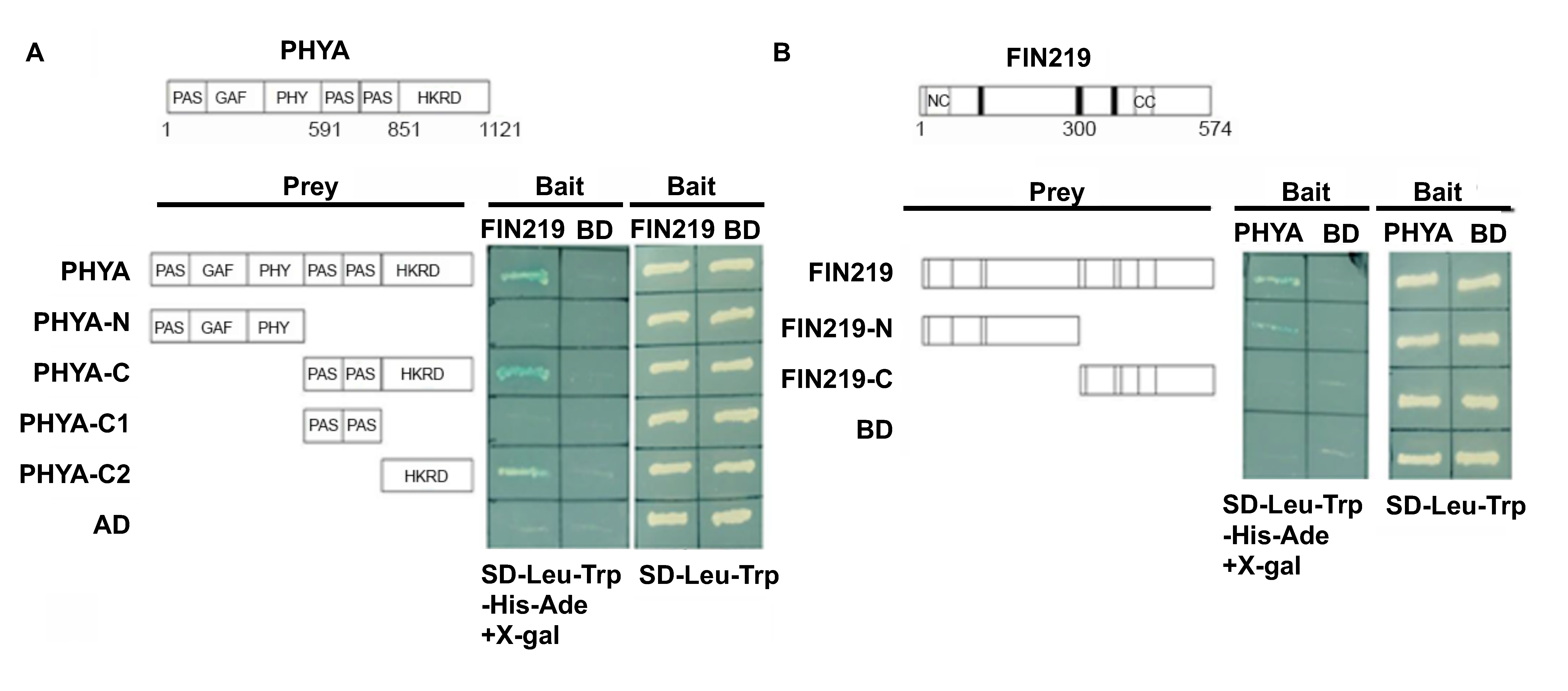

Supplement: S4 Fig — (A)Schematic diagrams of five different deletions of PHYA apoprotein as prey showed PHYA interaction with FIN219 as bait through its C-terminal domain. (B) Schematic diagrams of three different deletions of FIN219 as prey showed FIN219 interaction with PHYA as bait through its N-terminal domain. Yeast cells were transformed with PHYA-AD and FIN219-BD, grown on selective medium, and exhibited β-galactosidase activity. PAS: Per/Arndt/Sim domain; HKRD: histidine kinase-related domain; AD: active domain; NC: N-terminal coiled-coil domain; CC: C-terminal coiled-coil domain; BD: binding domain. (TIF) [file pgen.1010779.s004.tif]

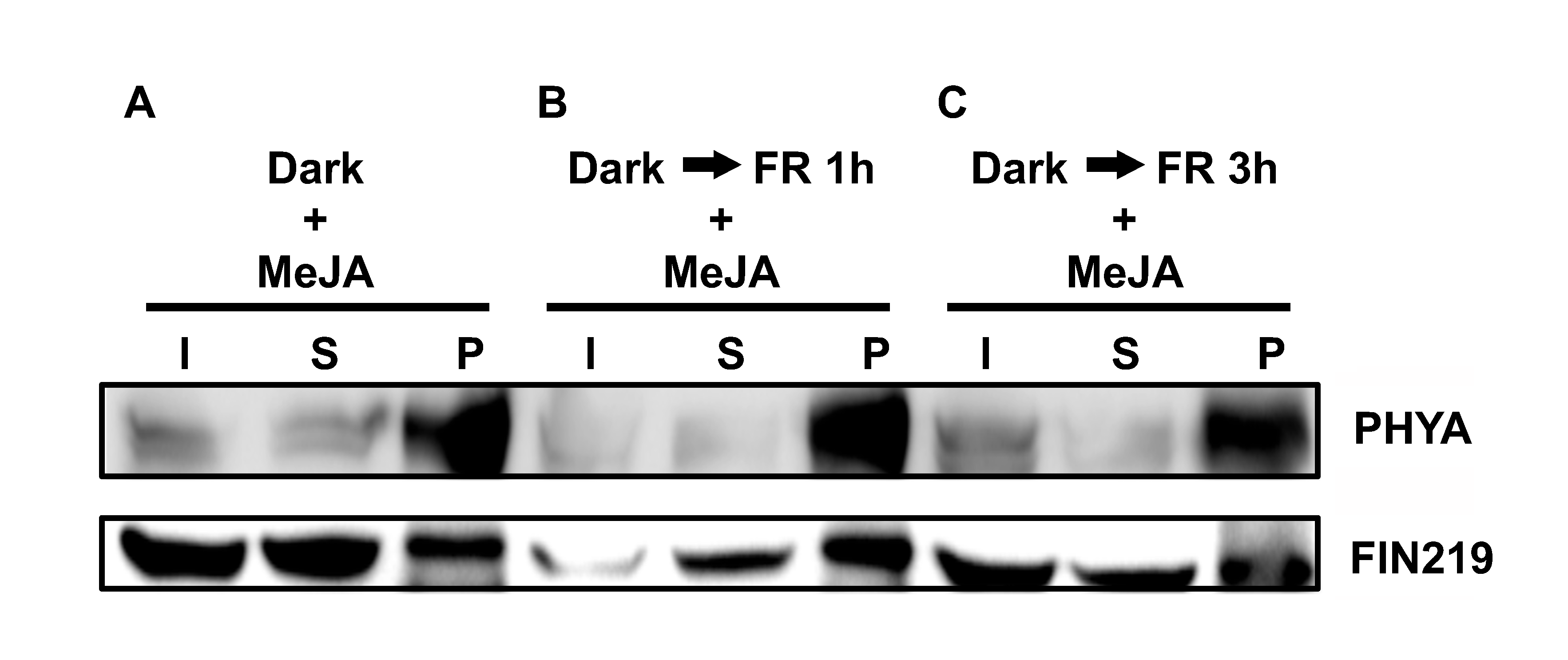

Supplement: S5 Fig — Col-0 seedlings were grown in the dark with 50 μM MeJA for 3 d (A) and then transferred to FR light (2 μmol m-2 s-1) for different times (B-C). Total proteins 2 mg extracted from the seedlings were immunoprecipitated with phyA monoclonal antibodies, then probed with PHYA and FIN219 monoclonal antibodies. I: input proteins; S: supernatants after immunoprecipitation; P: pellets after immunoprecipitation. (TIF) [file pgen.1010779.s005.tif]

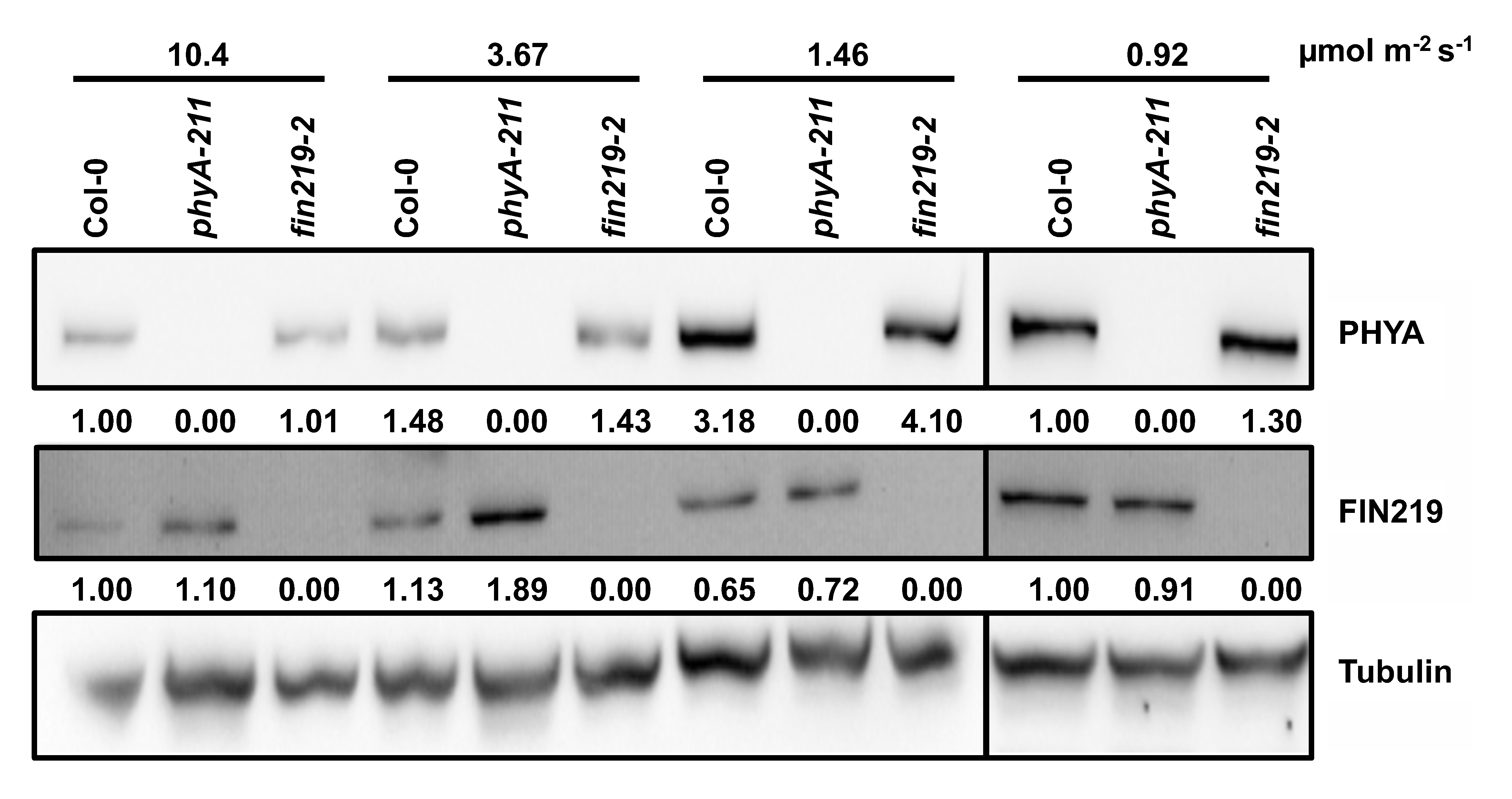

Supplement: S6 Fig — Protein gel blot analysis indicated that FIN219 levels in phyA mutant were differentially regulated by FR light fluences. The seedlings of Col-0, phyA-211 and fin219-2 were grown under different fluences of FR light for 3 days. The extracted proteins were subjected for Protein gel blot analysis. The primary antibodies were PHYA and FIN219 monoclonal antibodies. Tubulin was a loading control. The number below each blot represents the level of the indicated protein. The level of wild-type Col-0 was arbitrarily set to 1. (TIF) [file pgen.1010779.s006.tif]

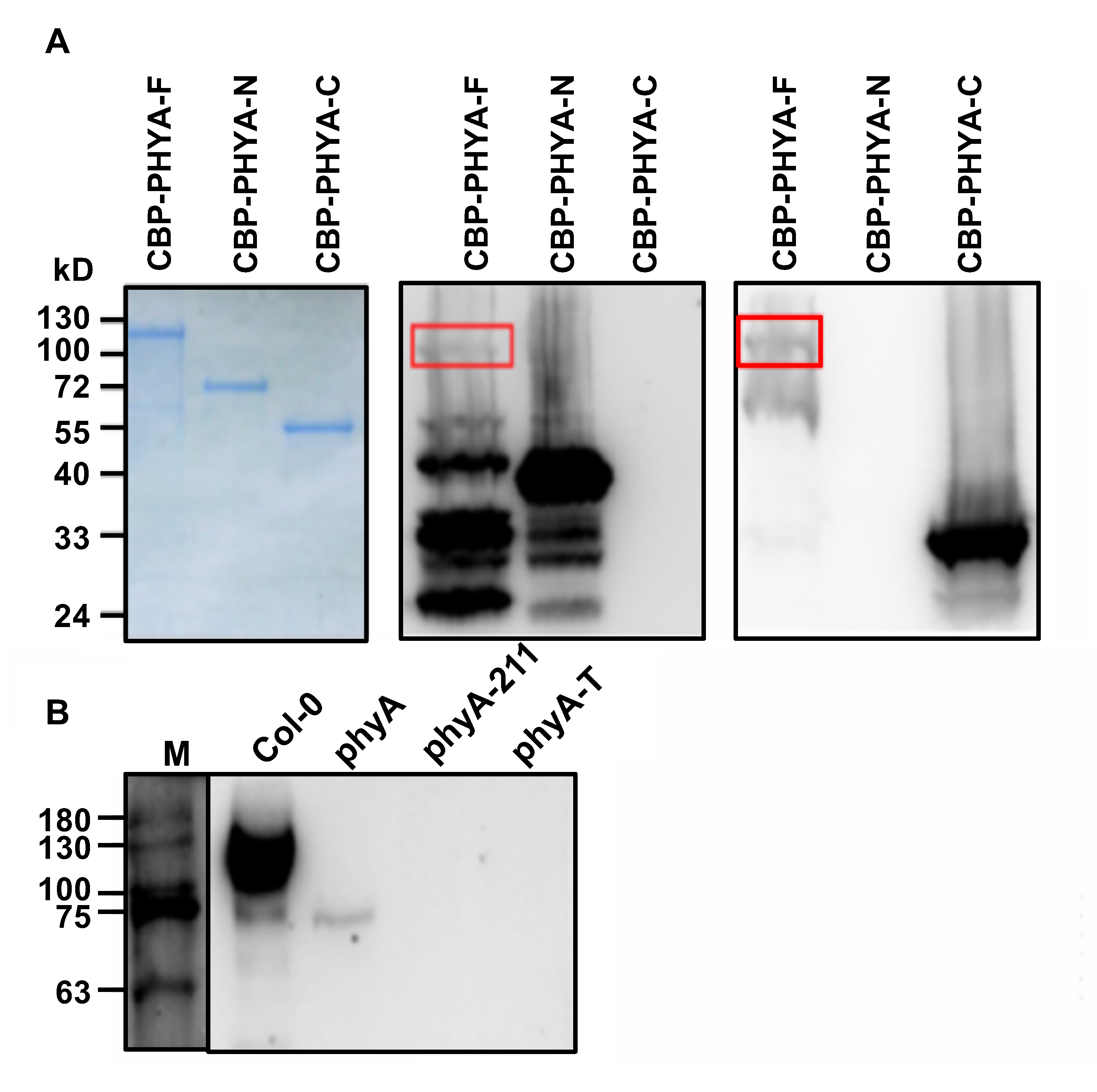

Supplement: S7 Fig — (A) Photograph of purified recombinant CBP-PHYA proteins. Purified recombinant proteins of the full-length and N-terminal and C-terminal domains of PHYA fused with a calmodulin binding peptide (CBP-PHYA-F, CBP-PHYA-N, and CBP-PHYA-C, respectively) were separated by 10% SDS-PAGE and stained with Coomassie blue dye (left panel). Protein gel blot analysis of recombinant CBP-PHYA proteins with different phyA monoclonal antibodies. PHYA monoclonal antibody #2 specifically recognized the full-length and N-terminus of PHYA (middle panel, with a longer running time of the gel) or PHYA monoclonal antibody #5 for the C-terminus of PHYA (right panel, with a longer running time of the gel). Dilution factor: 10,000X. (B) Protein gel blot analysis of PHYA protein detection specificity in seedlings of the indicated genotypes grown in the dark for 3 d. All phyA mutants were in a Columbia background. phyA: phyA-1; phyA-T: SALK_014575 mutants. Total protein (60 μg) was loaded in each lane and blots were probed with monoclonal antibody #2 against PHYA at dilution ratio 5000X. (TIF) [file pgen.1010779.s007.tif]

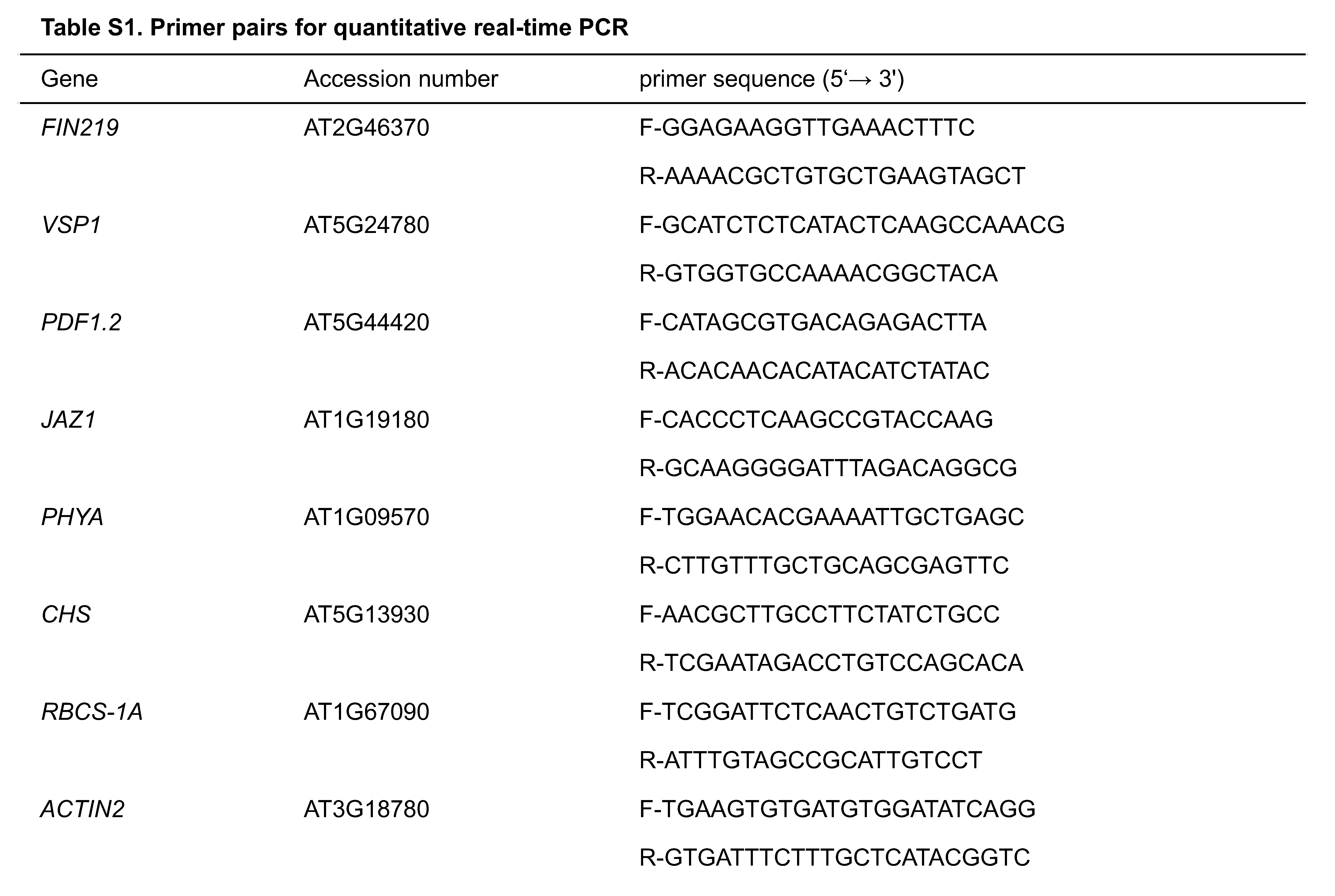

Supplement: S1 Table — (TIF) [file pgen.1010779.s008.tif]

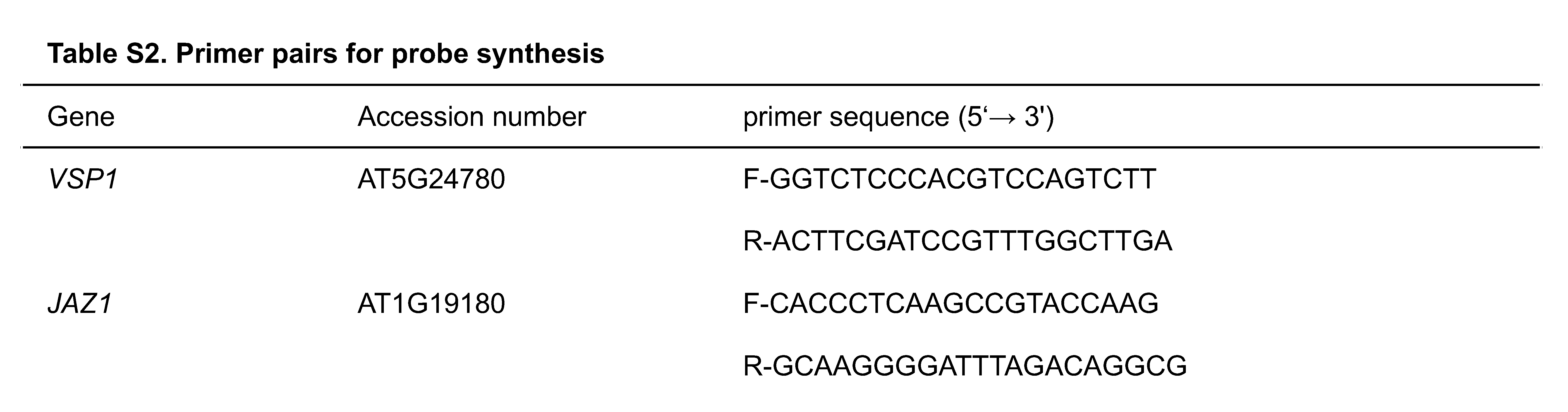

Supplement: S2 Table — (TIF) [file pgen.1010779.s009.tif]
